# Utility of SCORE2 risk algorithm for predicting life course accelerated frailty and physical function decline

**DOI:** 10.1002/jcsm.13165

**Published:** 2022-12-26

**Authors:** Chenglong Li, Yanjun Ma, Rong Hua, Fanfan Zheng, Wuxiang Xie

**Affiliations:** ^1^ Peking University Clinical Research Institute Peking University First Hospital Beijing China; ^2^ PUCRI Heart and Vascular Health Research Center Peking University, Shougang Hospital Beijing China; ^3^ School of Nursing, Peking Union Medical College Chinese Academy of Medical Sciences Beijing China

**Keywords:** Frailty trajectories, Physical function decline, Cardiovascular disease risk, Ageing, Healthy longevity

## Abstract

**Background:**

Frailty is a dynamic process that increases with ageing, while it remains unclear whether cardiovascular disease (CVD) risk algorithm could predict life course dynamic frailty trajectories, for example, the longitudinal patterns of how frailty evolves with time. We intended to examine the predictive utility of the Systemic Coronary Risk Estimation 2 (SCORE2) algorithm for life course accelerated frailty and physical function decline, in comparison with the precedent SCORE algorithm.

**Methods:**

Longitudinal data regarding accumulation of deficits frailty index (FI) and physical function (grip strength, gait speed, peak expiratory flow and timed chair rises) were drawn from the English Longitudinal Study of Ageing (ELSA) and Health and Retirement Study (HRS), two nationally representative cohorts with community‐dwelling adults aged ≥50 years. SCORE and SCORE2 were calculated at baselines following European Society of Cardiology guidelines. A group‐based trajectory modelling approach was used for identifying potential life course frailty trajectories, based on 14‐ and 12‐year FI data in the ELSA and HRS. Modified Poisson regression and linear mixed model were applied for analysing associations between SCORE2 with accelerated frailty trajectory and physical function decline, respectively. Receiver operating characteristic curve (ROC) analysis was conducted to evaluate predictive utility for accelerated frailty increase trajectory of SCORE and SCORE2, with the area under the curve (AUC) compared using the paired DeLong's test.

**Results:**

A total of 4834 participants from the ELSA and 7815 participants from the HRS were included (mean age: 64.0 ± 9.2 and 65.4 ± 9.9 years; men: 44.3% and 41.4%, respectively). Three frailty trajectories were consistently identified in both cohorts: (1) stable frailty increase (*n* = 3026 in ELSA and 4004 in HRS); (2) moderate frailty increase (*n* = 1325 in ELSA and 2955 in HRS); (3) accelerated frailty increase (*n* = 483 in ELSA and 856 in HRS). Each 10% increment in SCORE2 risk was associated with the higher risk of accelerated frailty increase (risk ratio [RR]: 3.58, 95% confidence interval [CI] [3.22, 3.98], *P* < 0.001 in ELSA; RR: 1.61, 95% CI [1.56, 1.67], *P* < 0.001 in HRS) and faster declines in all physical function measurements. SCORE2 algorithm showed good accuracy for predicting accelerated frailty increase (area under the curve [AUC] in ELSA: 0.759; HRS: 0.744), with better performance than the SCORE (AUC in ELSA: 0.729; HRS: 0.700) in both cohorts (*P* < 0.001 for comparison).

**Conclusions:**

SCORE2 algorithm could serve good utility for predicting life course accelerated frailty increase and physical function decline among community‐dwelling non‐frail adults aged ≥50 years.

## Introduction

Due to the ever‐expanding ageing population in a global range, attention to frailty is surging. As a multidimensional disorder, frailty is characterized by the deterioration in physiologic reserve and ability to maintain homeostasis and is associated with many adverse health outcomes, including mortality, hospitalization, and falls.^1^, ^2^ Many operational definitions are proposed, including the accumulation of deficit concept, the multidimensional concept, and the physical frailty phenotype concept.^3^ Despite the heterogeneity of definition, it is agreed by most experts that frailty is a dynamic process that increases with ageing.^1^ Considering the dynamic attributes of frailty, evaluating its life course trajectories, e.g. the longitudinal patterns of how frailty evolves with time, and identifying potentially modifiable risk factors could be significant for developing effective strategies of prevention.^4^ It also has been indicated that the frailty increase can be slowed or reversed by treatment, highlighting the need for early prediction to minimize potential adverse consequences.^5^ Traditional cardiovascular risk factors are identified as contributors to the elevated risk of frailty onset and progression.^1^ Nevertheless, these factors are generally correlated with each other, making it difficult to evaluate their single association with frailty. The cardiovascular disease (CVD) risk algorithm is developed to assess the overall cardiovascular risk burden and evaluate the risk of CVD in individuals, by incorporating traditional CVD risk factors.^6^ Despite the main utility of CVD risk management in practice, it has been found that some risk algorithms, including the Systemic Coronary Risk Estimation (SCORE), could also predict the incident frailty.^7^, ^8^ Nevertheless, these studies have focused on the dichotomized frailty status at certain time points, without considering the dynamic frailty trajectories in a longitudinal fashion. More importantly, the updated SCORE2 algorithm has been proposed for better CVD risk estimation, while few studies have investigated its predictive utility for life course frailty trajectories, in comparison with the precedent algorithms.^9^

Hence, we aim at identifying life course trajectories of accumulation of deficits frailty using a group‐based trajectory modelling (GBTM) approach. Then we will examine whether the SCORE2 risk algorithm could serve better predictive utility than its precedent SCORE algorithm in general community‐dwelling older adults. Given that decline in physical function is also deemed as another important phenotype of frailty increase, we incorporate it into the evaluation.^3^ Data came from two population‐based cohorts, namely, the English Longitudinal Study of Ageing (ELSA) and the Health and Retirement Study (HRS). We hypothesized that SCORE2 algorithm could serve the better utility for predicting life course accumulation of deficits frailty trajectories than the SCORE algorithm.

## Methods

### Study population

The HRS and the ELSA are two prospective and nationally representative cohorts of community‐dwelling adults aged ≥50 years in the United States and the United Kingdom. Access to details concerning the objectives, design and methods of the two cohorts can be found elsewhere.^10^, ^11^ Original survey datasets from the HRS and the ELSA are freely available to all bonafide researchers. Access to data can be obtained by visiting their websites (https://hrs.isr.umich.edu/about and https://www.elsa‐project.ac.uk/). The HRS was approved by the Institutional Reviewing Board at the University of Michigan and the National Institute on Aging (HUM00061128). The ELSA was approved by the London Multicentre Research Ethics Committee (MREC/01/2/91). Informed consent was obtained from all participants in both cohorts. The study was performed in accordance with the ethical standards laid down in the 1964 Declaration of Helsinki and its later amendments. Both cohorts conducted regular surveys, namely, wave, in a biennial fashion. We used 14‐year survey data from Wave 2 (the year 2004) to Wave 9 (the year 2018) in the ELSA and 12‐year data from Wave 8 (the year 2006) to Wave 14 (the year 2018) in the HRS for evaluation. Wave 2 of the ELSA and Wave 8 of the HRS were considered the baselines. The study timeline was presented in *Figure* *S1*. Participants were excluded if they met any of the following criteria: (1) with documented CVD or diabetes mellitus at baseline; (2) developed frailty at baseline; (3) without complete data for estimating 10‐year cardiovascular risk; (4) loss to follow‐up.

### Cardiovascular risk algorithm

The SCORE2 was calculated using individual values of age, sex, current smoking, systolic blood pressure (SBP), total cholesterol (TC) and high‐density lipoprotein cholesterol (HDL‐C) at baseline, according to the 2021 guidelines by the European Society of Cardiology (ESC).^9^ The calculated SCORE2 was further calibrated based on 4 clusters of countries (low, moderate, high and very high CVD risk).^9^ Based on published statistics, the United Kingdom was categorized as a low‐risk region while the United States was the high‐risk region.^9^, ^12^ Then we categorized participants into three risk categories (low‐to‐moderate risk, high risk and very high risk), according to risk classifications by ESC guidelines.^9^ The SCORE was also calculated using baseline values, including age, sex, current smoking, SBP and TC.^6^

### Frailty evaluation

Under the deficit accumulation frailty model by Rockwood, we followed a standard procedure to construct the frailty index (FI).^13^ After screening data availability, as well as accordance between the two cohorts, we included 29 items to construct the FI, including functional limitations (based on self‐reported difficulties in activities of daily living, instrumental activities of daily living, and other activities), self‐reported health status and alterations, components of depressive symptoms (based on the 8‐item version of the Center for Epidemiologic Studies Depression Scale), medical conditions (based on self‐reported diagnosis by physicians), and cognitive status (based on a combination of external physician diagnosis and cognition score). We defined these items according to previous studies.^14^ The 29 items constructing the FI were identical across different waves within each cohort and the two cohorts, with details for items definition shown in *Data* *S1* and *Table* *S1*. Each item represented a potential deficit, the score of which was further dichotomized or mapped into the 0.00 to 1.00 interval, with the value of 0.00 indicating the absence of a deficit and 1.00 indicating the maxim expression of a deficit. Then the continuous FI was calculated as the sum score of deficits divided by the number of items considered, for example, 29. The frailty status was defined as an FI ≥ 0.25.^2^

### Evaluation of physical function

Both the ELSA and the HRS conducted physical performance measurements at regular intervals, with standardized measurement protocols implemented by trained research nurses. For the ELSA, measurements including grip strength, peak expiratory flow, and timed five chair rises were conducted at Wave 2 (2004), Wave 4 (2008) and Wave 6 (2012) while measurement of the timed walk was conducted at each wave from Wave 2 (2004) to Wave 9 (2018). For the HRS, measurements including grip strength, peak expiratory flow and timed walk were all conducted at Wave 8 (2006), Wave 10 (2010), Wave 12 (2014) and Wave 14 (2018). The administered measurements had been verified with good validity in older adults.^15^ For the grip strength, the ELSA conducted three measurements, and the HRS conducted two measurements for each visit using a grip dynamometer, with records of the dominant hand used. We used the maximum measurement of the dominant hand for analysis, with a higher value indicating better performance. For the peak expiratory flow, both cohorts conducted three measurements for each visit, using a peak expiratory flow meter with a disposable mouthpiece. We used the maximum measurement for analysis, with a higher value indicating better performance. For the timed walk, both cohorts conducted two measurements for each visit. Each measurement recorded the seconds participants used to walk 244.0 cm (the ELSA) and 250.19 cm (the HRS) at a normal pace on a flat surface, with the use of a walking aid allowed. We used the minimum of the two measurements to calculate the gait speed (cm/s) of participants, with the higher value indicating better performance. The timed five chair rises implemented in the ELSA recorded the seconds participants used to stand up from a firm chair without using their arms for five times, with a lower value indicating better performance.

### Statistical analysis

The mean ± *SD* or the median with the interquartile range were used for descriptive statistics of continuous variables and numbers (percentage) for categorical variables. Differences in characteristics between groups were tested using the Student's *t* test, Wilcoxon rank test or *χ* ^2^ test. Based on the comparison results, we evaluated heterogeneity between the two cohorts and assessed appropriateness for pooling results.

We used the GBTM approach to evaluate the life course frailty trajectories, which was also applied by previous studies to investigate longitudinal trajectories of various indices.^16^ It assumed that the total population was consisted of several subpopulations with different longitudinal trajectories and used maximum likelihood estimation to identify participants sharing similar trajectories.^17^ We used SAS Proc Traj to fit GBTM models, with details described in *Data* *S1*. After identifying the potential accelerated frailty increase trajectory, we defined it as a binomial outcome, by combining the remaining identified trajectories as one category. Then we used the modified Poisson regression models to assess the association between baseline SCORE2 and subsequent risk of suffering accelerated frailty increase, with the robust sandwich estimator applied for calculation of risk ratio (RR) and the 95% confidence interval (CI). Receiver operating characteristic curve (ROC) analysis was conducted to evaluate predictive utility for accelerated frailty increase trajectory of SCORE and SCORE2, with the area under the curve (AUC) compared using the paired DeLong's test.

We used linear mixed models to assess longitudinal associations of SCORE2 with subsequent physical function decline. The intercept and slope of the time variable were fitted as random effects at the individual level to account for between‐participant differences at baseline and rates of changes. We used the Toeplitz covariance structure to model within‐participant correlations between repeated measurements, with Kenward–Roger adjustment to address the upward bias of test statistics for fixed model effects.^18^ Considering that the linear mixed models could appropriately handle dependent variable observations that were missing at random, no further imputation procedures were applied.^19^ More details concerning model construction are presented in the *Data* *S1*.

Several sensitivity analyses were conducted. First, we constructed a revised 26‐item FI after excluding hypertension, stroke and diabetes. Then we repeated our analysis regarding frailty trajectories. This analysis was to address the overlapping between SCORE2‐related variables and FI items, as well as examine whether the SCORE2 could predict frailty trajectories independently from cardiovascular events. Second, additional evaluations other than ROC analysis were conducted to comprehensively examine predictive utilities of the two algorithms.^20^ These evaluations included (1) the discrimination slope; (2) the continuous net reclassification improvement; (3) the integrated discrimination improvement; (4) the calibration analysis and Brier score (lower value indicates better accuracy); (5) the decision curve analysis. Third, we conducted an inverse probability weighting (IPW) analysis to handle the selection bias. Such approach was proposed to re‐weigh the included study samples while the analytical weight for each individual was calculated as the inverse of the probability of being included. We used binary logistic regression to estimate the inclusion probability.^21^ Absolute standardized mean differences were used for assessing the balance between included and excluded participants, with the Love plot selected for visualization. Weighted ROC analysis was conducted to incorporate the IPW weights. Fourth, to further examine the primary factor contributing to the long‐term accelerated frailty trajectory, we conducted two analyses. We first compared the baseline characteristics between different identified frailty trajectories, to give the hint regarding potential determinants. Then, we further controlled for different components of the SCORE2 risk algorithm in models separately. According to the previous literature, if the single component of the CVD risk algorithm largely explains the association between CVD risk and frailty, then further adjusting for the component would attenuate the observed association, compared with the unadjusted model.^22^ Fifth, to investigate the potential predictive value of SCORE2 for moderate frailty increase, we restricted the ROC analysis to individuals identified as stable and moderate frailty increase trajectories. Sixth, we stratified our analysis into individuals aged <65 years and ≥65 years to further examine whether SCORE2 could also hold predictive utility among middle‐aged and older adult populations, respectively.

Statistical analysis was conducted using SAS 9.4 (SAS Institute, Cary, NC, USA) and R language 3.6.2 (R Foundation, Vienna, Austria), with a two‐tailed alpha of 0.05 considered as statistically significant.

## Results

### Study participants

We included 4834 participants from the ELSA and 7815 participants from the HRS for analysis. The detailed inclusion process was shown in *Figure* *S2*.

Mean ages of included participants from the ELSA and the HRS were 64.0 ± 9.2 and 65.4 ± 9.9 years, respectively. The follow‐up duration of the study outcomes for the ELSA and HRS were 12.0 (8.0–14.0) and 12.0 (8.0–12.0) years, respectively. Men accounted for 44.3% and 41.4% of the cohorts, respectively. The mean SCORE2 (%) in the ELSA were 7.0 ± 4.8 and 12.4 ± 10.7 in the HRS, respectively. Categorized using the SCORE2, participants of very high risk accounted for 10% of included individuals in the ELSA, while 39.3% in the HRS. As shown in *Table* *1*, differences existed in most baseline characteristics between the two cohorts. Considering the observed heterogeneity between the two cohorts, we did not perform the pooled analysis.

**Table 1:** Baseline characteristics of participants from two independent cohorts

Characteristics^a^	ELSA (*N* = 4834)	HRS (*N* = 7815)	*P* for difference^b^
Age (years)	64.0 ± 9.2	65.4 ± 9.9	<0.001
Age range (years)	63.0 (57.0–70.0)	65.0 (57.0–72.0)	<0.001
Men (%)	2141 (44.3%)	3235 (41.4%)	<0.001
Follow‐up duration (years)	12.0 (8.0–14.0)	12.0 (8.0–12.0)	<0.001
White (%)	4760 (98.5%)	6642 (85.0%)	<0.001
Living alone (%)	1215 (25.1%)	2082 (26.6%)	0.064
Current smoking (%)	624 (12.9%)	1022 (13.1%)	0.805
Drinking ≥3 times per week (%)	1967 (40.7%)	1726 (22.1%)	<0.001
Physical Exercise (%)	4223 (87.4%)	6517 (83.4%)	<0.001
Height (m)	1.7 ± 0.1	1.7 ± 0.1	0.741
Weight (kg)	75.7 ± 14.9	79.0 ± 17.1	<0.001
BMI (kg/m^2^)	27.4 ± 4.5	28.7 ± 5.6	<0.001
SBP (mmHg)	134.5 ± 18.5	130.2 ± 20.3	<0.001
DBP (mmHg)	76.0 ± 10.8	79.8 ± 11.3	<0.001
TC (mmol/L)	6.0 ± 1.1	5.6 ± 1.5	<0.001
HDL‐C (mmol/L)	1.6 ± 0.4	1.6 ± 0.5	<0.001
SCORE risk	8.4 ± 9.7	8.2 ± 9.3	0.178
SCORE2 risk	7.0 ± 4.8	12.4 ± 10.7	<0.001
SCORE2 risk categories (%)			<0.001
Low‐to‐moderate risk	2196 (45.4%)	2008 (25.7%)	
High risk	2153 (44.5%)	2739 (35.0%)	
Very high risk	485 (10.0%)	3068 (39.3%)	
Grip strength (kg)	33.3 ± 11.2	33.1 ± 11.1	0.313
Timed 5 chair rises (s)	11.0 ± 3.5	—	—
Peak expiratory flow (litres/min)	352.0 ± 140.1	379.2 ± 129.3	<0.001
Gait speed (cm/s)	99.9 ± 27.4	86.9 ± 23.4	<0.001
Hypertension (%)	1543 (31.9%)	3306 (42.3%)	<0.001
Chronic lung diseases (%)	166 (3.4%)	332 (4.2%)	0.025
Cancer (%)	291 (6.0%)	868 (11.1%)	<0.001
Frailty index	0.08 (0.04–0.12)	0.09 (0.04–0.15)	<0.001

Abbreviations: BMI, body mass index; DBP, diastolic blood pressure; ELSA, English Longitudinal Study of Aging; HDL‐C, high‐density lipoprotein cholesterol; HRS, Health and Retirement Study; SBP, systolic blood pressure; SCORE, Systemic Coronary Risk Estimation; SCORE2, Systemic Coronary Risk Estimation 2; TC, total cholesterol.

^a^ Data are presented as mean ± *SD*, *n* (%) or median (Quartile 1–Quartile 3).

^b^ *P* value reported for differences between two cohorts using *t* test, *χ* ^2^ test or Wilcoxon rank test. “—” represents no measurements were conducted.

### Identified life course accumulation of deficits frailty trajectories

**Figure 1:**
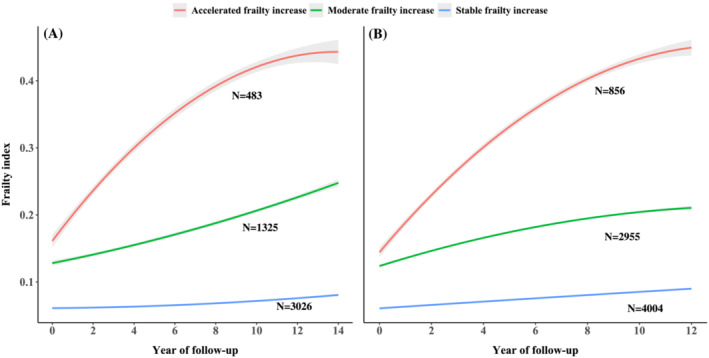
Identified dynamic frailty trajectories using the group‐based trajectory modelling approach in two independent cohorts. Panel (*A*): frailty trajectories in the English Longitudinal Study of Ageing (ELSA) cohort; Panel (*B*): frailty trajectories in the Health and Retirement Study (HRS) cohort.

Based on the GBTM approach, three potential frailty increase trajectories were consistently identified in both cohorts, shown in *Figure* *1*. The identified trajectories included (1) stable frailty increase (*n* = 3026 in ELSA and 4004 in HRS), with consistently stable growth in FI; (2) moderate frailty increase (*n* = 1325 in ELSA and 2955 in HRS), with moderate growth in FI; (3) accelerated frailty increase (*n* = 483 in ELSA and 856 in HRS), with comparatively steep growth in FI.

### Predictive utility of SCORE2 algorithm for frailty trajectories

As presented in *Table* *2*, for both cohorts, each 10% elevated SCORE2 risk was consistently associated with a higher risk of suffering accelerated frailty increase (RR: 3.58, 95% CI [3.22, 3.98], *P* < 0.001 in ELSA; RR: 1.61, 95% CI [1.56, 1.67], *P* < 0.001 in HRS).

**Table 2:** Association between SCORE2 risk and accelerated frailty increase in two independent cohorts

SCORE2 risk	ELSA (*N* = 4834)			HRS (*N* = 7815)		
	Events/total	RR (95% CI)^a^	*P*	Events/total	RR (95% CI)^a^	*P*
Low‐to‐moderate risk	89/2196	Reference	/	63/2008	Reference	/
High risk	245/2153	2.81 [2.22, 3.55]	<0.001	198/2739	2.30 [1.75, 3.04]	<0.001
Very high risk	149/485	7.58 [5.94, 9.67]	<0.001	595/3068	6.18 [4.80, 7.96]	<0.001
Test for linear trend^b^	—	2.75 [2.44, 3.08]	<0.001	—	2.56 [2.30, 2.85]	<0.001
Per 10% increment^c^	—	3.58 [3.22, 3.98]	<0.001	—	1.61 [1.56, 1.67]	<0.001

Abbreviations: CI, confidence interval; ELSA, English Longitudinal Study of Aging; HRS, Health and Retirement Study; RR, risk ratio; SCORE2, Systemic Coronary Risk Estimation 2.

^a^ RR was estimated using modified Poisson regression models.

^b^ Performed by treating SCORE2 risk categories as a numerical variable.

^c^ Estimated as the RR for SCORE2 risk in percentage.

**Figure 2:**
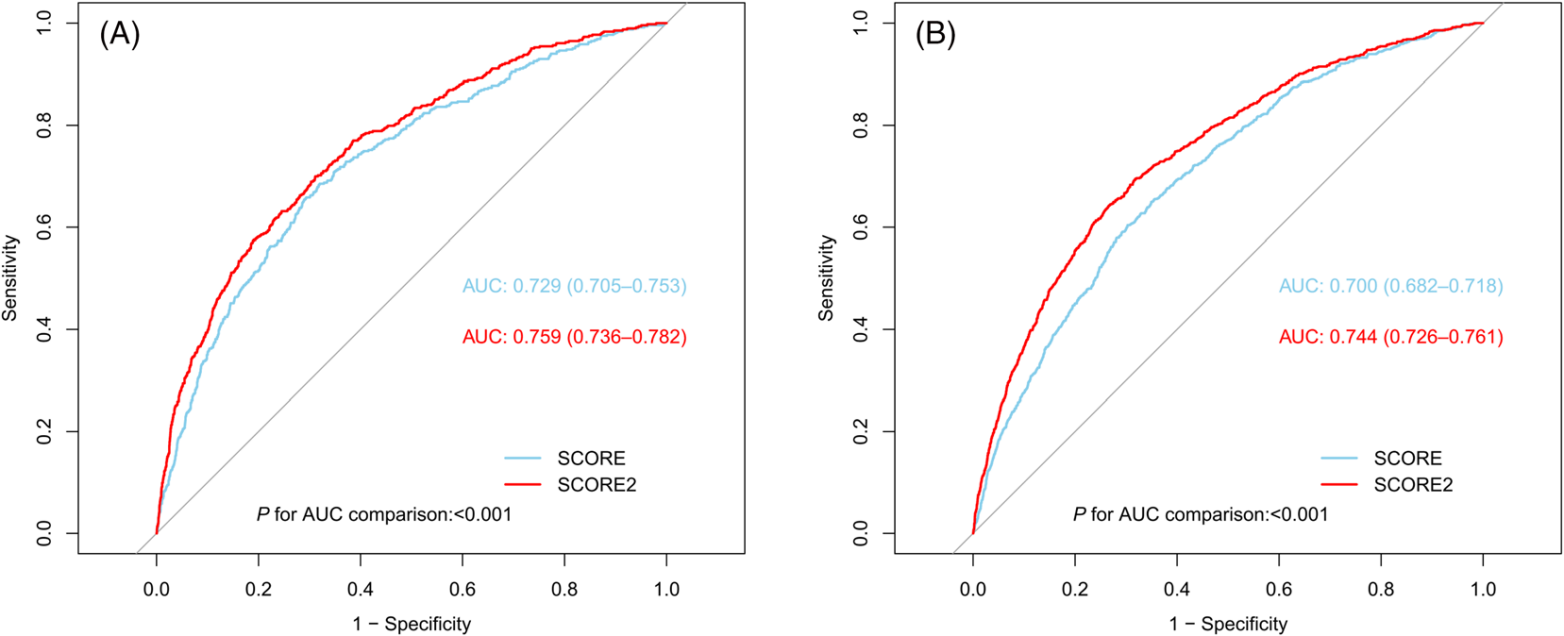
ROC analysis comparing the performance of predicting accelerated frailty increase by SCORE and SCORE2 in two independent cohorts. Panel (*A*): ROC analysis in the ELSA; Panel (*B*): ROC analysis in the HRS. ROC, receiver operating characteristic curve; AUC, area under the curve; ELSA, English Longitudinal Study of Aging; HRS: Health and Retirement Study; SCORE, Systemic Coronary Risk Estimation; SCORE2, Systemic Coronary Risk Estimation 2.

*Figure* *2* showed results for evaluation of predictive accuracy for accelerated frailty increase of SCORE and SCORE2. As observed in both cohorts, both the SCORE (AUC in ELSA: 0.729; AUC in HRS: 0.700) and SCORE2 (AUC in ELSA: 0.759; AUC in HRS: 0.744) showed good abilities for predicting further accelerated frailty increase, with the SCORE2 algorithm consistently presenting better performance in overall accuracy than the SCORE algorithm (*P* < 0.001 for AUC comparison in both cohorts).

#### Predictive utility of SCORE2 algorithm for physical function decline

As presented in *Table* *3*, for both cohorts, elevated SCORE2 risk was consistently associated with accelerated physical function decline. Compared with participants of low‐to‐moderate risk, those of high and very high risk suffered accelerated declines in all objective physical function measurements, as shown in both cohorts. A significant linear dose–response association pattern was also identified in both cohorts, shown in *Table* *3*.

**Table 3:** Association between SCORE2 risk and rate of change in physical function in two independent cohorts

SCORE2 risk	ELSA (*N* = 4834)		HRS (N = 7815)	
	Beta (95% CI)^a^	*P*	Beta (95% CI)^a^	*P*
Rate of change in grip strength (kg/year)				
Low‐to‐moderate risk	Reference	/	Reference	/
High risk	−0.221 [−0.324, −0.118]	<0.001	−0.211 [−0.295, −0.126]	<0.001
Very high risk	−0.443 [−0.639, −0.247]	<0.001	−0.347 [−0.434, −0.260]	<0.001
Test for linear trend^b^	−0.230 [−0.310, −0.150]	<0.001	−0.175 [−0.219, −0.132]	<0.001
Per 10% increment^c^	−0.374 [−0.491, −0.256]	<0.001	−0.133 [−0.171, −0.094]	<0.001
Rate of change in peak expiratory flow (litres/min/year)				
Low‐to‐moderate risk	Reference	/	Reference	/
High risk	−2.219 [−3.730, −0.709]	0.004	−2.526 [−3.494, −1.558]	<0.001
Very high risk	−9.902 [−12.910, −6.890]	<0.001	−4.577 [−5.572, −3.583]	<0.001
Test for linear trend	−3.551 [−4.746, −2.356]	<0.001	−2.337 [−2.835, −1.840]	<0.001
Per 10% increment	−6.260 [−8.055, −4.465]	<0.001	−1.842 [−2.276, −1.408]	<0.001
Rate of change in gait speed (cm/s/year)				
Low‐to‐moderate risk	Reference	/	Reference	/
High risk	−0.786 [−0.975, −0.598]	<0.001	−0.583 [−0.929, −0.238]	0.002
Very high risk	−1.404 [−1.793, −1.015]	<0.001	−0.946 [−1.289, −0.602]	<0.001
Test for linear trend	−0.698 [−0.849, −0.547]	<0.001	−0.380 [−0.516, −0.244]	<0.001
Per 10% increment	−1.369 [−1.594, −1.144]	<0.001	−0.518 [−0.617, −0.418]	<0.001
Rate of change in timed 5 chair rises (s/year)				
Low‐to‐moderate risk	Reference	/	Reference	/
High risk	0.084 [0.045, 0.123]	<0.001	—	—
Very high risk	0.114 [0.029, 0.198]	0.008	—	—
Test for linear trend	0.070 [0.038, 0.101]	<0.001	—	—
Per 10% increment	0.151 [0.102, 0.200]	<0.001	—	—

Abbreviations: CI, confidence interval; ELSA, English Longitudinal Study of Aging; HRS, Health and Retirement Study; SCORE2, Systemic Coronary Risk Estimation 2.

^a^ Beta coefficient was estimated using linear mixed models.

^b^ Performed by treating SCORE2 risk categories as a numerical variable.

^c^ Estimated as the beta coefficient for SCORE2 risk in percentage.

### Sensitivity analyses

After repeating the primary analysis based on the revised 26‐item FI, our findings concerning frailty trajectories were not materially changed, as observed in both cohorts (*Table* S2, *Figures S3* and *S4*). When applying other indices than the AUC, we found that the SCORE2 continues to show better predictive accuracy than the SCORE, with significant differences detected in both cohorts (*Table* *S3*). SCORE2 also consistently presented better calibration accuracy (*Figures* *S5* and *S6*) and more net benefit (*Figures* *S7* and *S8*) than the SCORE.

As shown in *Figures* *S9* and *S10*, after the IPW weighting, differences between included and excluded participants were diminished in both cohorts, compared with the original unweighted samples. And SCORE2 continued to show better overall accuracy than the SCORE in the weighted ROC analysis (*Figures* *S11* and *S12*).

Based on displayed results in *Tables* *S4* and *S5*, those individuals identified as accelerated frailty increase were generally older, had higher FI levels at baseline and had higher CVD risk levels, as consistent in both cohorts. After further controlling for each component of SCORE2, respectively, significant attenuation in associations was only observed when the age variable was included in models, as shown in both cohorts (*Table* *S6*).

As shown in *Figure* *S13*, the SCORE2 also showed utility for predicting the moderate frailty increase from the stable frailty in both cohorts (AUC in ELSA: 0.663; AUC in HRS: 0.672), with better performance in overall accuracy than the SCORE algorithm (*P* < 0.001 for AUC comparison in both cohorts). When restricted to the younger population aged <65 years, the SCORE2 did not achieve an accuracy as good as in the overall population (AUC in ELSA: 0.615; AUC in HRS: 0.627), as shown in *Figure* *S14*. By contrast, the SCORE2 maintained predictive utility among older pollution aged ≥65 years (AUC in ELSA: 0.677; AUC in HRS: 0.681), as shown in *Figure* *S15*.

## Discussion

Based on 14‐year survey data from the ELSA and 12‐year survey data from the HRS, we found that the elevated SCORE2 risk was consistently associated with life course frailty trajectories and physical function decline among community‐dwelling non‐frail adults aged ≥50 years. And the SCORE2 algorithm slightly showed better performance than the SCORE algorithm for predicting life course accelerated frailty increase, as mutually verified in both cohorts. To our knowledge, this is the first prospective study to evaluate the potential predictive utility of the SCORE2 risk algorithm for life course frailty trajectories and physical function decline, with consistent findings from two population‐based cohorts of community‐dwelling non‐frail adults.

Several previous studies evaluated the association between CVD risk and frailty, in both cross‐sectional and prospective manners. In a cross‐sectional study of 8175 participants aged ≥50 years in the United Kingdom, researchers found that elevated SCORE risk was associated with higher FI and odds of being frailty (defined as an FI ≥ 0.25).^23^ In a Whitehall II cohort study of 3895 participants aged 45–69 years in the United Kingdom, researchers explored the predictive utility by CVD risk for future frailty defined using the 5‐item Fried frailty scale.^7^ They used the Framingham CVD risk score, the Framingham coronary heart disease risk score, the Framingham stroke risk score and the SCORE algorithms to estimate the CVD risk.^7^ After a 10‐year follow‐up, they found that all four algorithms could predict incident frailty and the association pertained after excluding incident CVD cases.^7^ In another study based on the ELSA cohort, researchers also found that the Framingham CVD risk score could predict the incident frailty.^22^ Also observed in another pooled study of two cohorts, researchers found that elevated CVD risk score, defined by American Heart Association, also could predict future frailty status.^8^ Despite the shared findings, these studies generally focused on the frailty status instead of the frailty trajectories. As the frailty increase is a dynamic process, identifying relative contributors to the accelerated frailty increase could be crucial for early identification and prevention.^1^ Our study indicated that the elevated SCORE2 risk was consistently associated with life course accelerated frailty increase, indicating the clinical utility of applying the SCORE2 algorithm to identify those at risk of suffering accelerated frailty increase in community settings.

In addition to frailty increase, we also identified that the elevated SCORE2 risk could predict accelerated physical function decline evaluated using objective measurements. Some previous studies also presented similar findings. In a cohort study of 1441 participants aged 60 and older in Sweden, researchers investigated the association between Framingham CVD risk score and the risk of future limitations in walking speed during a 9‐year follow‐up.^24^ They found elevated Framingham CVD risk score was associated with a higher risk of developing walking limitations, and faster decline in walking speed in participants younger than 78.^24^ Another study pooled 37 cohort studies from 24 countries also found that elevated burden of non‐communicable diseases risk factors also was associated with lost in physical function.^25^

The major implication of our findings was the SCORE2 risk algorithm could serve as a useful tool for predicting life course accelerated frailty increase and physical function decline among community‐dwelling non‐frail adults aged ≥50 years. In a prospective study based on electronic health records of community‐dwelling adults in the United Kingdom, researchers used the latent growth mixture modelling to evaluate the life course frailty trajectories measured using the FI.^26^ In similarity with our findings, they also identified three distinct trajectories of frailty, including the stable growth trajectory, the moderate increase trajectory and the rapidly rising trajectory.^26^ And they further found that compared with participants of the stable growth of frailty, those of the moderately increasing frailty were associated with a 65% increase in mortality, while the rapidly rising trajectory was associated with a 180% increase in mortality.^26^ Such findings supported the dynamic nature of frailty and, more importantly, the prognostic value of frailty trajectories for identifying the risk of mortality.^26^ Likewise, in another study involving community‐dwelling adults, researchers identified that participants belonging to trajectories of increasing frailty scores or showing consistently higher frailty levels suffered an increased risk of mortality and other adverse outcomes, compared with those of maintaining low or showing decreases in frailty levels.^27^ In addition to frailty trajectories, the decline in physical function measurements, including gait speed, grip and timed chair rises, was also found to be associated with increased risk of CVD events and mortality.^28^, ^29^ These contributions further extended the clinical relevance of our findings.

Interestingly, we found that the association between SCORE2 and accelerated frailty was mainly explained by chronological age other than traditional and modifiable CVD risk factors. We also found that further excluding the CVD onset from FI calculation did not significantly influence the predictive performance of SCORE2 for life course accelerated frailty. These findings supported the older age as the main factor contributing to the accelerated course of frailty. We also found that the SCORE2 algorithm performed better among the older adults aged ≥65 years, compared with the younger population aged <65 years. This was noteworthy. Given that older age could represent a worse clinical course of frailty, such findings indicated that SCORE2 could be more suitable for predicting life course accelerated frailty among older people aged ≥ 65 years, rather than the younger population. In the context of the ever‐increasing aged population, the indication could be crucial for developing strategies targeting the promotion of healthy ageing and longevity in a global range.

Our study had several strengths. First, based on 14‐year survey data from the ELSA and 12‐year survey data from the HRS, we were able to evaluate the life course dynamic frailty trajectories based on multiple repeated measurements and investigate the role of the SCORE2 risk algorithm in accelerated frailty increase prediction. The GBTM approach we used could efficiently incorporate repeated measurements of FI at multiple timepoints, thus addressing limitations by only considering frailty status at a single time‐point. Second, we also incorporated objective measurements of physical function into consideration, with multiple indices for evaluating the decline in physical function. Third, our findings were robust, with generally consistent findings verified in two independent cohorts. Finally, our study population was representative of community‐dwelling adults aged ≥50 years, with large sample sizes.

We also acknowledged several important limitations. First, the majority of our participants were of White ethnicity, hence restricting generalization to other ethnicities. Second, we excluded a considerable number of participants from the analysis, resulting in potential selection bias. Although we conducted a sensitivity analysis to address the issue, our findings still could be biased and therefore should be interpreted in caution. Third, most of the selected items for FI calculation were irreversible deficits; thus, the possibility of improvement or recovery from frailty was precluded in our study. This could lead to biased findings, as some chronic conditions or deficits could be improved or cured. Finally, due to the nature of observational studies, we could not eliminate the impact by residual confounding, which impedes further steps towards conclusively defining causal relationships.^30^

In summary, we found that the SCORE2 risk algorithm could serve a good predictive utility for life course frailty trajectories and physical function decline among community‐dwelling non‐frail adults aged ≥50 years. In addition to serving as a tool for CVD risk estimation, applying SCORE2 risk algorithm in community settings for risk prediction and stratification of life course accelerated frailty and physical function decline might be as relevant for the current practice.

## Conflict of interest

None declared.

## Funding

The present study was supported by the National Natural Science Foundation of China (project no. 81974490). The funder had no role in the study design; the collection, analysis and interpretation of data; the writing of the manuscript; or the decision to submit the article for publication.

## Supporting information


**Table S1.** The components of constructed 29‐item frailty index.
Table S2. Association between SCORE2 risk and accelerated frailty increase in two independent cohorts, based on the modified 26‐item (excluding hypertension, stroke, and diabetes) frailty index.
Table S3. Additional indices evaluating predictive ability of SCORE and SCORE2 for accelerated frailty increase in 2 independent cohorts.
Table S4. Baseline characteristics of participants of different frailty trajectories in the ELSA cohort.
Table S5. Baseline characteristics of participants of different frailty trajectories in the HRS cohort.
Table S6. Association between SCORE2 risk and accelerated frailty increase in two independent cohorts, further controlling for components of SCORE2.
Figure S1. Study timeline and design.
Figure S2. Participants selection diagram.
Figure S3. Identified dynamic frailty trajectories using the group‐based trajectory modelling approach in two independent cohorts, based on the modified 26‐item (excluding hypertension, stroke, and diabetes) frailty index.
Figure S4. ROC analysis comparing the performance of predicting accelerated frailty increase by SCORE and SCORE2 in two independent cohorts, based on the modified 26‐item (excluding hypertension, stroke, and diabetes) frailty index.
Figure S5. Calibration plot comparing the performance of predicting accelerated frailty increase by SCORE and SCORE2 in the ELSA cohort.
Figure S6. Calibration plot comparing the performance of predicting accelerated frailty increase by SCORE and SCORE2 in the HRS cohort.
Figure S7. Decision curve analysis for assessing the risk threshold of predicting accelerated frailty increase by SCORE and SCORE2 in the ELSA cohort.
Figure S8. Decision curve analysis for assessing the risk threshold of predicting accelerated frailty increase by SCORE and SCORE2 in the HRS cohort.
Figure S9. Love plot assessing balance between included and excluded participants in the ELSA cohort, before and after the IPW weighting.
Figure S10. Love plot assessing balance between included and excluded participants in the HRS cohort, before and after the IPW weighting.
Figure S11. Weighted ROC analysis comparing the performance of predicting accelerated frailty increase by SCORE and SCORE2 in the ELSA cohort, using the IPW as sample weights.
Figure S12. Weighted ROC analysis comparing the performance of predicting accelerated frailty increase by SCORE and SCORE2 in the HRS cohort, using the IPW as sample weights.
Figure S13. ROC analysis comparing the performance of predicting moderate frailty increase by SCORE and SCORE2 in two independent cohorts.
Figure S14. ROC analysis comparing the performance of predicting accelerated frailty increase by SCORE and SCORE2 in two independent cohorts, restricted to individuals aged < 65 years.
Figure S15. ROC analysis comparing the performance of predicting accelerated frailty increase by SCORE and SCORE2 in two independent cohorts, restricted to individuals aged ≥ 65 years.
Data S1. Supplemental Methods.
