# Supplementary material for: Utility of SCORE2 risk algorithm for predicting life course accelerated frailty and physical function decline
Source: J Cachexia Sarcopenia Muscle. 2022 Dec 26;14(1):596–605. doi: 10.1002/jcsm.13165 (PMC9891950; doi:10.1002/jcsm.13165)
Supplement: Supplementary file 1 — Table S1. The components of constructed 29‐item frailty index. Table S2. Association between SCORE2 risk and accelerated frailty increase in two independent cohorts, based on the modified 26‐item (excluding hypertension, stroke, and diabetes) frailty index. Table S3. Additional indices evaluating predictive ability of SCORE and SCORE2 for accelerated frailty increase in 2 independent cohorts. Table S4. Baseline characteristics of participants of different frailty trajectories in the ELSA cohort. Table S5. Baseline characteristics of participants of different frailty trajectories in the HRS cohort. Table S6. Association between SCORE2 risk and accelerated frailty increase in two independent cohorts, further controlling for components of SCORE2. Figure S1. Study timeline and design. Figure S2. Participants selection diagram. Figure S3. Identified dynamic frailty trajectories using the group‐based trajectory modelling approach in two independent cohorts, based on the modified 26‐item (excluding hypertension, stroke, and diabetes) frailty index. Figure S4. ROC analysis comparing the performance of predicting accelerated frailty increase by SCORE and SCORE2 in two independent cohorts, based on the modified 26‐item (excluding hypertension, stroke, and diabetes) frailty index. Figure S5. Calibration plot comparing the performance of predicting accelerated frailty increase by SCORE and SCORE2 in the ELSA cohort. Figure S6. Calibration plot comparing the performance of predicting accelerated frailty increase by SCORE and SCORE2 in the HRS cohort. Figure S7. Decision curve analysis for assessing the risk threshold of predicting accelerated frailty increase by SCORE and SCORE2 in the ELSA cohort. Figure S8. Decision curve analysis for assessing the risk threshold of predicting accelerated frailty increase by SCORE and SCORE2 in the HRS cohort. Figure S9. Love plot assessing balance between included and excluded participants in the ELSA cohort, before and after the IPW weightin [file JCSM-14-596-s001.docx]

**SUPPLEMENTAL MATERIAL**

**Table of contents**

[**Supplemental Methods** 3](#_Toc109822273)

[**Frailty Assessment** 3](#_Toc109822274)

[**Statistical Analyses** 4](#_Toc109822275)

[**References** 5](#_Toc109822276)

[**Supplemental Tables** 6](#_Toc109822277)

[**Supplemental Figures** 6](#_Toc109822278)

**Supplemental Methods**

**Frailty Assessment**

The 29 items used to construct the FI included functional limitations (based on self-reported difficulties in activities of daily living, instrumental activities of daily living, and other activities), self-reported health status and alterations, components of depressive symptoms (based on 8-item version of the Center for Epidemiologic Studies Depression Scale), medical conditions (based on self-reported diagnosis by physicians), and cognitive status (based on a combination of external physician diagnosis and cognition score). These items came from self-reported information by study participants and objective measurements. The functional limitations were derived from the Activities of Daily Living (ADLs) and Instrumental Activities of Daily Living (IADLs), as well as other self-reported subjective activities limitations. Self-reported health status and alterations were derived using the self-rating of participants’ overall health status. Components of depressive symptoms were extracted from 8-item version of the Center for Epidemiologic Studies Depression Scale, which included 8 questions concerning indicators of participants’ feelings much of the time over the week prior to the interview, with each question consisting of 2 response categories (yes or no). The medical conditions were extracted from self-reported physician diagnosis of relevant diseases, with confirmation procedure applied at each wave for both cohorts. Such procedure was applied to help participants confirm whether they had been diagnosed of the disease at previous wave. The cognitive status was measured based on a combination of self-reported diagnosis and cognition score. We defined dementia cases using either a self-reported physician diagnosis or an alternative approach based on cognition and functionality scores. For the HRS, we used a cognition summary score from 0 to 27, with a cutoff point of 6 or less defined as dementia and 7 to 11 defined as cognitive impairment [1]. The cognition summary score comprised the domains of memory and executive function [1]. For the ELSA, we defined dementia as a coexistence of cognitive and functional impairment. Cognitive impairment was defined as a score that was 1.5 SD below the mean of the population stratified by educational background [2]. Functional impairment was defined as difficulty in performing one or more activities of daily living, including bathing, eating, dressing, getting in/out of bed, and walking across a room. These approaches have been validated using data from the same HRS and ELSA cohorts, respectively [1,2,3].

**Statistical Analyses**

SCORE2 risk metrics were analyzed via 3 approaches:1) SCORE2 risk categories, fitted as a categorical variable, with the low-ro-moderate risk as reference level; 2) SCORE2 risk categories, fitted as a numerical variable to perform linear trend test; 3) SCORE2 risk in percentage, to reflect associations on a scale of per 10% risk increment.

Based on the 14-year and 12-year repeated measurements of FI in ELSA and HRS, we used the GBTM to evaluate long-term frailty increase trajectories. The GBTM approach can fit non-monotonic trajectories and support multiple trajectory shapes including linear, quadratic and cubic. It also allows specification of number of trajectory groups before fitting the model. We selected number of groups from 3 to 5 and compared model fit statistics of the Bayesian information criterion (BIC) of different trajectory models to determine the most optimal number of trajectory groups. Then, we determined that modeling 3 trajectory groups was appropriate for frailty trajectories modeling, as consistent in both cohorts.

We further evaluated different trajectory shapes for each trajectory group by testing the null hypothesis that the shape parameter for the group equals zero. We also used graphics of trajectory group means to help determine which shape best fit each trajectory group. After the procedure, we determined that the best 3-group trajectory model consisted of 3 quadratic trajectories. Then the estimated trajectory groups membership was included as the dependent variable for further multivariate analysis.

To assess longitudinal associations between SCORE2 and physical function decline, we constructed linear mixed models included: SCORE2 risk metrics, time, SCORE2 risk metrics × time, and covariates. The coefficient of the interaction term SCORE2 risk metrics × time can reflect associations between SCORE2 risk metrics and change rate in objective physical function measurements with time.

**References**

1. Crimmins EM, Kim JK, Langa KM, Weir DR. Assessment of cognition using surveys and neuropsychological assessment: the Health and Retirement Study and the Aging, Demographics, and Memory Study. *J Gerontol B Psychol Sci Soc Sci* 2011;**66 Suppl 1**:i162-71.

2. Ahmadi-Abhari S, Guzman-Castillo M, Bandosz P *et al.* Temporal trend in dementia incidence since 2002 and projections for prevalence in England and Wales to 2040: modelling study. *BMJ* 2017;**358**:j2856.

3. Grasset L, Glymour MM, Yaffe K *et al.* Association of traumatic brain injury with dementia and memory decline in older adults in the United States. *Alzheimers Dement* 2020;**16**:853–861.

**Supplemental Tables**

[**Table S1.** The components of constructed 29-item frailty index.](#_Toc109388184)

[**Table S2.** Association between SCORE2 risk and accelerated frailty increase in two independent cohorts, based on the modified 26-item (excluding hypertension, stroke, and diabetes) frailty index.](#_Toc109388185)

[**Table S3.** Additional indices evaluating predictive ability of SCORE and SCORE2 for accelerated frailty increase in 2 independent cohorts.](#_Toc109388186)

[**Table S4.** Baseline characteristics of participants of different frailty trajectories in the ELSA cohort.](#_Toc109388184)

[**Table S5.** Baseline characteristics of participants of different frailty trajectories in the HRS cohort.](#_Toc109388185)

[**Table S6.** Association between SCORE2 risk and accelerated frailty increase in two independent cohorts, further controlling for components of SCORE2.](#_Toc109388186)

**Supplemental Figures**

**Figure S1.** Study timeline and design.

**Figure S2.** Participants selection diagram.

**Figure S3.** Identified dynamic frailty trajectories using the group-based trajectory modeling approach in two independent cohorts, based on the modified 26-item (excluding hypertension, stroke, and diabetes) frailty index.

**Figure S4.** ROC analysis comparing the performance of predicting accelerated frailty increase by SCORE and SCORE2 in two independent cohorts, based on the modified 26-item (excluding hypertension, stroke, and diabetes) frailty index.

**Figure S5.** Calibration plot comparing the performance of predicting accelerated frailty increase by SCORE and SCORE2 in the ELSA cohort.

**Figure S6.** Calibration plot comparing the performance of predicting accelerated frailty increase by SCORE and SCORE2 in the HRS cohort.

**Figure S7.** Decision curve analysis for assessing the risk threshold of predicting accelerated frailty increase by SCORE and SCORE2 in the ELSA cohort.

**Figure S8.** Decision curve analysis for assessing the risk threshold of predicting accelerated frailty increase by SCORE and SCORE2 in the HRS cohort.

**Figure S9.** Love plot assessing balance between included and excluded participants in the ELSA cohort, before and after the IPW weighting.

**Figure S10.** Love plot assessing balance between included and excluded participants in the HRS cohort, before and after the IPW weighting.

**Figure S11.** Weighted ROC analysis comparing the performance of predicting accelerated frailty increase by SCORE and SCORE2 in the ELSA cohort, using the IPW as sample weights.

**Figure S12.** Weighted ROC analysis comparing the performance of predicting accelerated frailty increase by SCORE and SCORE2 in the HRS cohort, using the IPW as sample weights.

**Figure S13.** ROC analysis comparing the performance of predicting moderate frailty increase by SCORE and SCORE2 in two independent cohorts.

**Figure S14.** ROC analysis comparing the performance of predicting accelerated frailty increase by SCORE and SCORE2 in two independent cohorts, restricted to individuals aged < 65 years.

**Figure S15.** ROC analysis comparing the performance of predicting accelerated frailty increase by SCORE and SCORE2 in two independent cohorts, restricted to individuals aged ≥ 65 years.

**Table S1. The components of constructed 29-item frailty index.**

| **Item number ^a^** | **Item definition ^b^** | **Scoring ^c^** |
| --- | --- | --- |
| 1 | Self-reported difficulties in bathing because of a physical, mental, emotional or memory problem. | Yes=1.00; No=0.00 |
| 2 | Self-reported difficulties in dressing because of a physical, mental, emotional or memory problem. | Yes=1.00; No=0.00 |
| 3 | Self-reported difficulties in getting in/out of bed because of a physical, mental, emotional or memory problem. | Yes=1.00; No=0.00 |
| 4 | Self-reported difficulties in walking around the house because of a physical, mental, emotional or memory problem. | Yes=1.00; No=0.00 |
| 5 | Self-reported difficulties in eating because of a physical, mental, emotional or memory problem. | Yes=1.00; No=0.00 |
| 6 | Self-reported difficulties in finishing daily activities of using the toilet because of a physical, mental, emotional or memory problem. | Yes=1.00; No=0.00 |
| 7 | Total number (0-5) of self-reported difficulties in finishing daily activities including bathing, dressing, eating, getting in/out of bed, walking around the house. | Any difficulties=1.00; No difficulties=0.00 |
| 8 | Self-reported difficulties in shopping because of a physical, mental, emotional or memory problem. | Yes=1.00; No=0.00 |
| 9 | Self-reported difficulties in preparing hot meal because of a physical, mental, emotional or memory problem. | Yes=1.00; No=0.00 |
| 10 | Self-reported difficulties in taking prescribed medications because of a physical, mental, emotional or memory problem. | Yes=1.00; No=0.00 |
| 11 | Self-reported difficulties in managing money because of a physical, mental, emotional or memory problem. | Yes=1.00; No=0.00 |
| 12 | Self-reported difficulties in getting up from a chair because of a physical, mental, emotional or memory problem. | Yes=1.00; No=0.00 |
| 13 | Self-reported difficulties in climbing several flights of stairs because of a physical, mental, emotional or memory problem. | Yes=1.00; No=0.00 |
| 14 | Self-reported difficulties in lifting or carrying weights over 10 pounds because of a physical, mental, emotional or memory problem. | Yes=1.00; No=0.00 |
| 15 | Self-reported difficulties in walking one block because of a physical, mental, emotional or memory problem. | Yes=1.00; No=0.00 |
| 16 | Self-reported rating of health status. | Poor=1.00; Fair=0.75; Good=0.50; Very Good=0.25; Excellent=0.00 |
| 17 | Change in self-reported rating of health status. | Worse=1.00; Better/Same=0.00 |
| 18 | Feeling that everything is an effort much of time. | Yes=1.00; No=0.00 |
| 19 | Feeling depressed much of time. | Yes=1.00; No=0.00 |
| 20 | Feeling happy much of time. | No=1.00; Yes=0.00 |
| 21 | Feeling lonely much of time. | Yes=1.00; No=0.00 |
| 22 | Feeling that could not get going much of time. | Yes=1.00; No=0.00 |
| 23 | Self-reported diagnosis of hypertension by physician. | Yes=1.00; No=0.00 |
| 24 | Self-reported diagnosis of stroke by physician. | Yes=1.00; No=0.00 |
| 25 | Self-reported diagnosis of cancer by physician. | Yes=1.00; No=0.00 |
| 26 | Self-reported diagnosis of diabetes by physician. | Yes=1.00; No=0.00 |
| 27 | Self-reported diagnosis of arthritis by physician. | Yes=1.00; No=0.00 |
| 28 | Self-reported diagnosis of chronic lung disease by physician. | Yes=1.00; No=0.00 |
| 29 | Cognitive status, based on combination of self-reported diagnosis, and cognition score. | Dementia=1.00; Cognitive impairment but not demented=0.50; Cognitive healthy=0.00 |

^a^ An unified 29-item frailty index was utilized in both the ELSA and the HRS.

^b^ Item definition was based on self-reported information or objective measurements or both.

^c^ The frailty index was calculated as the sum of scoring divided by the total number of items.

**Table S2.** **Association between SCORE2 risk and accelerated frailty increase in two independent cohorts, based on the modified 26-item (excluding hypertension, stroke, and diabetes) frailty index.**

| **SCORE2 risk ^a^** | **ELSA (N=4834)** | | | **HRS (N=7815)** | | |
| --- | --- | --- | --- | --- | --- | --- |
|  | **Events/Total** | **RR (95% CI) ^b^** | ***P*** | **Events/Total** | **RR (95% CI) ^b^** | ***P*** |
| Low-to-moderate risk | 90/2196 | Reference | / | 59/2008 | Reference | / |
| High risk | 233/2153 | 2.64 (2.09, 3.34) | <0.001 | 174/2739 | 2.16 (1.62, 2.89) | <0.001 |
| Very high risk | 144/485 | 7.24 (5.67, 9.25) | <0.001 | 527/3068 | 5.85 (4.49, 7.61) | <0.001 |
| Test for linear trend ^c^ | - | 2.70 (2.39, 3.04) | <0.001 | - | 2.52 (2.25, 2.82) | <0.001 |
| Per 10% increment ^d^ | - | 3.53 (3.16, 3.93) | <0.001 | - | 1.62 (1.56, 1.68) | <0.001 |

**^a^** SCORE2: Systemic Coronary Risk Estimation 2; ELSA: English Longitudinal Study of Aging; HRS: Health and Retirement Study; RR: risk ratio; CI: confidence interval.

**^b^** RR was estimated using modified Poisson regression models.

^c^ Performed by treating SCORE2 risk categories as a numerical variable.

^d^ Estimated as the beta coefficient for SCORE2 risk in percentage.

**Table S3. Additional indices evaluating predictive ability of SCORE and SCORE2 for accelerated frailty increase in 2 independent cohorts.**

| **Model ^a^** | **SCORE** | **SCORE2** | ***P* for comparison ^b^** |
| --- | --- | --- | --- |
| **ELSA (N=4834)** | | | |
| Discrimination slope | 0.061 | 0.109 | <0.001 |
| NRI (continuous) | 1 [Reference] | 0.749 (0.659, 0.839) | <0.001 |
| Absolute IDI (95% CI) | 1 [Reference] | 0.048 (0.039, 0.056) | <0.001 |
| **HRS (N=7815)** | | | |
| Discrimination slope | 0.040 | 0.091 | <0.001 |
| NRI (continuous) | 1 [Reference] | 0.673 (0.600, 0.746) | <0.001 |
| Absolute IDI (95% CI) | 1 [Reference] | 0.051 (0.043, 0.059) | <0.001 |

**^a^** NRI: net reclassification improvement; IDI: integrated discrimination improvement; ELSA: English Longitudinal Study of Aging; HRS: Health and Retirement Study.

**^b^** The *Z* test was applied for comparison of indices.

**Table S4. Baseline characteristics of participants of different frailty trajectories in the ELSA cohort.**

| **Characteristics ^a^** | **Stable frailty**  **(N=3026)** | **Moderate frailty**  **(N=1325)** | **Accelerated frailty**  **(N=483)** | ***P* for difference ^b^** |
| --- | --- | --- | --- | --- |
| Age (years) | 61.3±7.7 | 67.0±9.2 | 72.7±10.0 | <0.001 |
| Men (%) | 1449 (47.9%) | 511 (38.6%) | 181 (37.5%) | <0.001 |
| Follow-up duration (years) | 14.0 (8.0-14.0) | 12.0 (8.0-14.0) | 8.0 (4.0-12.0) | <0.001 |
| White (%) | 2982 (98.5%) | 1304 (98.4%) | 474 (98.1%) | 0.779 |
| Living alone (%) | 595 (19.7%) | 403 (30.4%) | 217 (44.9%) | <0.001 |
| Current smoking (%) | 362 (12.0%) | 179 (13.5%) | 83 (17.2%) | 0.005 |
| Drinking ≥ 3 times per week (%) | 1383 (45.7%) | 456 (34.4%) | 128 (26.5%) | <0.001 |
| Physical Exercise (%) | 2773 (91.6%) | 1109 (83.7%) | 341 (70.6%) | <0.001 |
| BMI (kg/m^2^) | 26.9±4.1 | 28.3±4.8 | 28.2±5.5 | <0.001 |
| SBP (mmHg) | 132.2±17.4 | 137.9±19.3 | 139.3±20.7 | <0.001 |
| DBP (mmHg) | 76.1±10.5 | 76.3±11.0 | 74.1±11.5 | <0.001 |
| TC (mmol/L) | 6.0±1.1 | 6.0±1.3 | 5.9±1.1 | 0.061 |
| HDL-C (mmol/L) | 1.6±0.4 | 1.5±0.4 | 1.5±0.4 | <0.001 |
| SCORE risk | 6.3±7.1 | 10.6±11.2 | 15.9±13.7 | <0.001 |
| SCORE2 risk | 5.7±3.7 | 8.3±5.1 | 11.6±6.1 | <0.001 |
| Grip strength (kg) | 35.5±11.0 | 30.6±10.6 | 27.2±10.5 | <0.001 |
| Timed 5 chair rises (s) | 10.2±3.0 | 12.0±3.6 | 13.6±4.3 | <0.001 |
| Peak expiratory flow (liters/min) | 378.9±138.6 | 320.8±129.1 | 265.8±125.6 | <0.001 |
| Gait speed (cm/s) | 107.9±26.0 | 94.9±25.9 | 80.6±23.6 | <0.001 |
| Hypertension (%) | 706 (23.3%) | 602 (45.4%) | 235 (48.7%) | <0.001 |
| Chronic lung diseases (%) | 53 (1.8%) | 65 (4.9%) | 48 (9.9%) | <0.001 |
| Cancer (%) | 122 (4.0%) | 122 (9.2%) | 47 (9.7%) | <0.001 |
| Frailty index | 0.04 (0.02-0.09) | 0.12 (0.08-0.16) | 0.16 (0.09-0.21) | <0.001 |

ELSA: English Longitudinal Study of Aging; HRS: Health and Retirement Study; BMI: body mass index; SBP: systolic blood pressure; DBP: diastolic blood pressure; TC: total cholesterol; HDL-C: High-density lipoprotein cholesterol; SCORE: Systemic Coronary Risk Estimation; SCORE2: Systemic Coronary Risk Estimation 2.

**^a^** Data are presented as mean ± SD, n (%), or median (quartile 1–quartile 3).

**^b^** P value reported for differences between trajectory groups using analysis of variance, chi-square test, or Kruskal-Wallis test. “-” represents no measurements were conducted.

**Table S5. Baseline characteristics of participants of different frailty trajectories in the HRS cohort.**

| **Characteristics ^a^** | **Stable frailty**  **(N=4004)** | **Moderate frailty**  **(N=2955)** | **Accelerated frailty**  **(N=856)** | ***P* for difference ^b^** |
| --- | --- | --- | --- | --- |
| Age (years) | 62.2±9.0 | 67.6±9.2 | 72.9±10.0 | <0.001 |
| Men (%) | 1737 (43.4%) | 1168 (39.5%) | 330 (38.6%) | 0.001 |
| Follow-up duration (years) | 12.0 (10.0-12.0) | 12.0 (8.0-12.0) | 8.0 (6.0-12.0) | <0.001 |
| White (%) | 3486 (87.1%) | 2446 (82.8%) | 710 (82.9%) | <0.001 |
| Living alone (%) | 848 (21.2%) | 904 (30.6%) | 330 (38.6%) | <0.001 |
| Current smoking (%) | 477 (11.9%) | 405 (13.7%) | 140 (16.4%) | 0.001 |
| Drinking ≥ 3 times per week (%) | 981 (24.5%) | 600 (20.3%) | 145 (16.9%) | <0.001 |
| Physical Exercise (%) | 3537 (88.3%) | 2348 (79.5%) | 632 (73.8%) | <0.001 |
| BMI (kg/m^2^) | 28.0±5.0 | 29.6±6.0 | 28.5±6.1 | <0.001 |
| SBP (mmHg) | 127.2±19.0 | 132.7±20.6 | 135.5±22.7 | <0.001 |
| DBP (mmHg) | 79.6±10.9 | 80.1±11.6 | 79.5±12.4 | 0.172 |
| TC (mmol/L) | 5.7±1.6 | 5.5±1.5 | 5.4±1.5 | <0.001 |
| HDL-C (mmol/L) | 1.7±0.5 | 1.6±0.5 | 1.6±0.5 | <0.001 |
| SCORE risk | 6.0±7.1 | 9.4±9.9 | 14.0±12.5 | <0.001 |
| SCORE2 risk | 9.0±8.1 | 14.3±10.8 | 21.6±14.1 | <0.001 |
| Grip strength (kg) | 35.2±11.2 | 31.5±10.5 | 28.3±10.3 | <0.001 |
| Timed 5 chair rises (s) | . | . | . | . |
| Peak expiratory flow (liters/min) | 410.9±127.2 | 357.2±121.3 | 306.7±120.4 | <0.001 |
| Gait speed (cm/s) | 93.5±22.4 | 85.3±22.4 | 74.3±23.0 | <0.001 |
| Hypertension (%) | 1184 (29.6%) | 1653 (55.9%) | 469 (54.8%) | <0.001 |
| Chronic lung diseases (%) | 44 (1.1%) | 197 (6.7%) | 91 (10.6%) | <0.001 |
| Cancer (%) | 251 (6.3%) | 476 (16.1%) | 141 (16.5%) | <0.001 |
| Frailty index | 0.05 (0.03-0.09) | 0.13 (0.09-0.17) | 0.16 (0.12-0.20) | <0.001 |

ELSA: English Longitudinal Study of Aging; HRS: Health and Retirement Study; BMI: body mass index; SBP: systolic blood pressure; DBP: diastolic blood pressure; TC: total cholesterol; HDL-C: High-density lipoprotein cholesterol; SCORE: Systemic Coronary Risk Estimation; SCORE2: Systemic Coronary Risk Estimation 2.

**^a^** Data are presented as mean ± SD, n (%), or median (quartile 1–quartile 3).

**^b^** P value reported for differences between trajectory groups using analysis of variance, chi-square test, or Kruskal-Wallis test. “-” represents no measurements were conducted.

**Table S6. Association between SCORE2 risk and accelerated frailty increase in two independent cohorts, further controlling for components of SCORE2.**

| **SCORE2 risk ^a^** | **ELSA (N=4834)** | | | **HRS (N=7815)** | | |
| --- | --- | --- | --- | --- | --- | --- |
|  | **Events/Total** | **RR (95% CI) ^b^** | ***P*** | **Events/Total** | **RR (95% CI) ^b^** | ***P*** |
| **SCORE2 risk, unadjusted** |  |  |  |  |  |  |
| Low-to-moderate risk | 89/2196 | Reference | / | 63/2008 | Reference | / |
| High risk | 245/2153 | 2.81 (2.22, 3.55) | <0.001 | 198/2739 | 2.30 (1.75, 3.04) | <0.001 |
| Very high risk | 149/485 | 7.58 (5.94, 9.67) | <0.001 | 595/3068 | 6.18 (4.80, 7.96) | <0.001 |
| **Further adjusted for age** |  |  |  |  |  |  |
| Low-to-moderate risk | 89/2196 | Reference | / | 63/2008 | Reference | / |
| High risk | 245/2153 | 1.23 (0.94, 1.61) | 0.127 | 198/2739 | 1.32 (0.99, 1.77) | 0.062 |
| Very high risk | 149/485 | 1.40 (0.96, 2.04) | 0.081 | 595/3068 | 1.98 (1.45, 2.72) | <0.001 |
| **Further adjusted for smoking** |  |  |  |  |  |  |
| Low-to-moderate risk | 89/2196 | Reference | / | 63/2008 | Reference | / |
| High risk | 245/2153 | 2.82 (2.22, 3.58) | <0.001 | 198/2739 | 2.31 (1.75, 3.04) | <0.001 |
| Very high risk | 149/485 | 7.67 (5.94, 9.91) | <0.001 | 595/3068 | 6.20 (4.80, 8.00) | <0.001 |
| **Further adjusted for SBP** |  |  |  |  |  |  |
| Low-to-moderate risk | 89/2196 | Reference | / | 63/2008 | Reference | / |
| High risk | 245/2153 | 3.01 (2.37, 3.82) | <0.001 | 198/2739 | 2.36 (1.78, 3.12) | <0.001 |
| Very high risk | 149/485 | 8.71 (6.67, 11.37) | <0.001 | 595/3068 | 6.58 (5.03, 8.62) | <0.001 |
| **Further adjusted for TC** |  |  |  |  |  |  |
| Low-to-moderate risk | 89/2196 | Reference | / | 63/2008 | Reference | / |
| High risk | 245/2153 | 2.82 (2.23, 3.57) | <0.001 | 198/2739 | 2.28 (1.73, 3.01) | <0.001 |
| Very high risk | 149/485 | 7.53 (5.90, 9.60) | <0.001 | 595/3068 | 6.07 (4.71, 7.81) | <0.001 |
| **Further adjusted for HDL-C** |  |  |  |  |  |  |
| Low-to-moderate risk | 89/2196 | Reference | / | 63/2008 | Reference | / |
| High risk | 245/2153 | 2.84 (2.23, 3.62) | <0.001 | 198/2739 | 2.33 (1.76, 3.07) | <0.001 |
| Very high risk | 149/485 | 7.68 (5.97, 9.88) | <0.001 | 595/3068 | 6.29 (4.87, 8.12) | <0.001 |

**^a^** SCORE2: Systemic Coronary Risk Estimation 2; ELSA: English Longitudinal Study of Aging; HRS: Health and Retirement Study; RR: risk ratio; CI: confidence interval; SBP: systolic blood pressure; TC: total cholesterol; HDL-C: High-density lipoprotein cholesterol.

**^b^** RR was estimated using modified Poisson regression models.


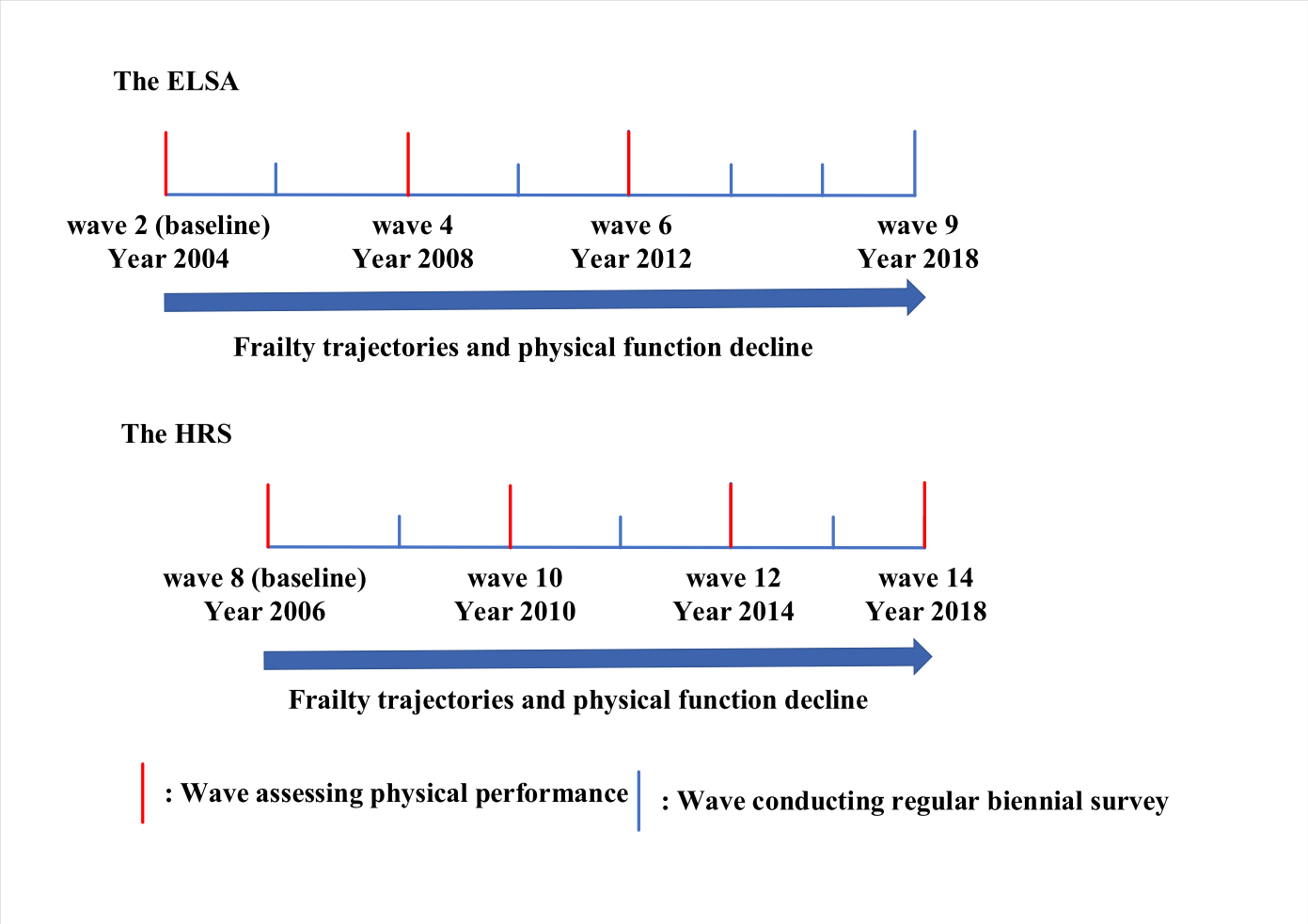


**Figure S1. Study timeline and design.**

The vertical lines represent each separate survey for the ELSA and HRS, with length between lines indicating interval between two consecutive survey in years. Red lines represent waves conducting both regular biennial survey and physical function measurements. Blue lines represent waves only conducting regular biennial survey.

ELSA: English Longitudinal Study of Ageing.

HRS: Health and Retirement Study.


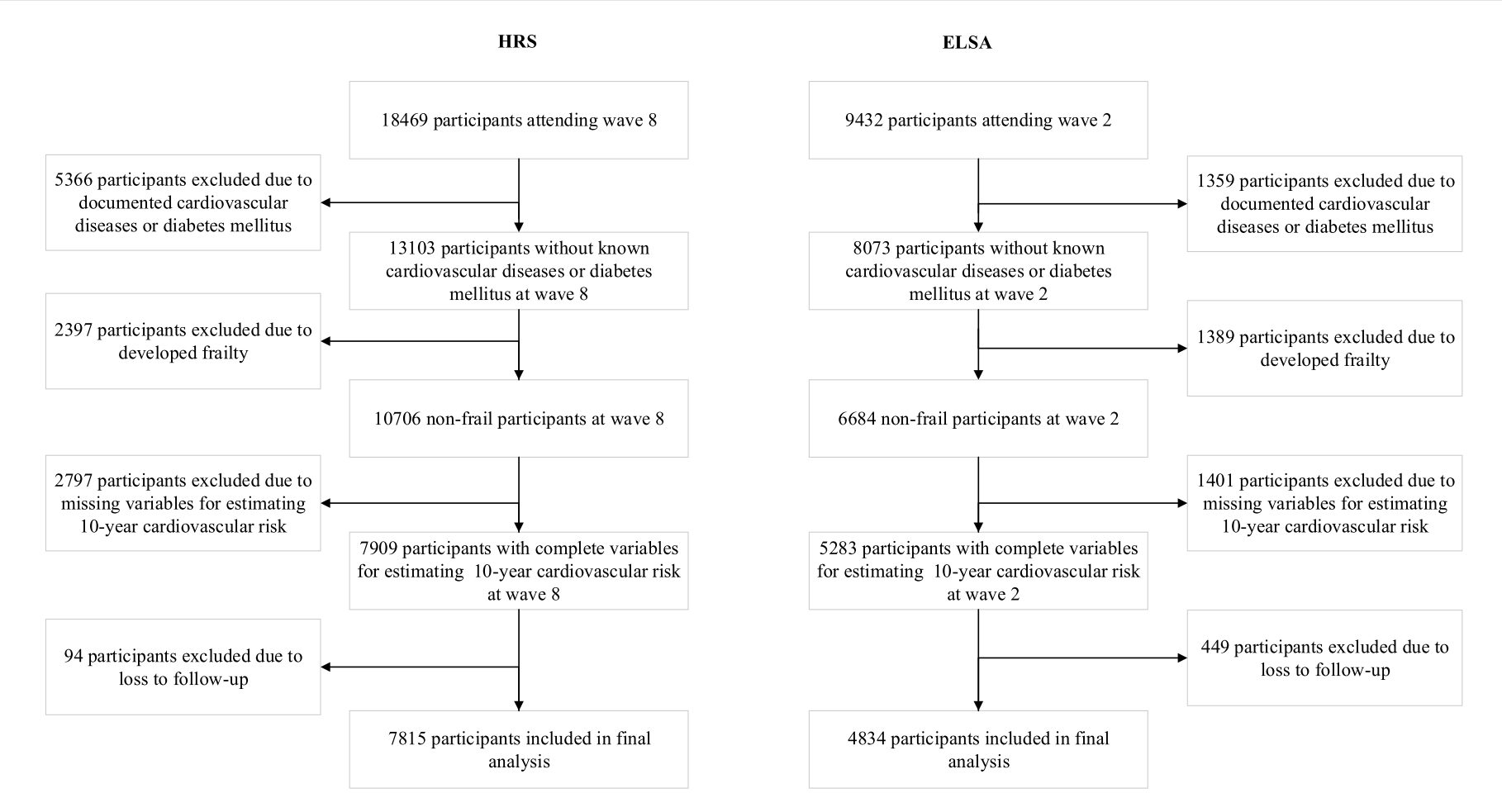


**Figure S2. Participants selection diagram.**


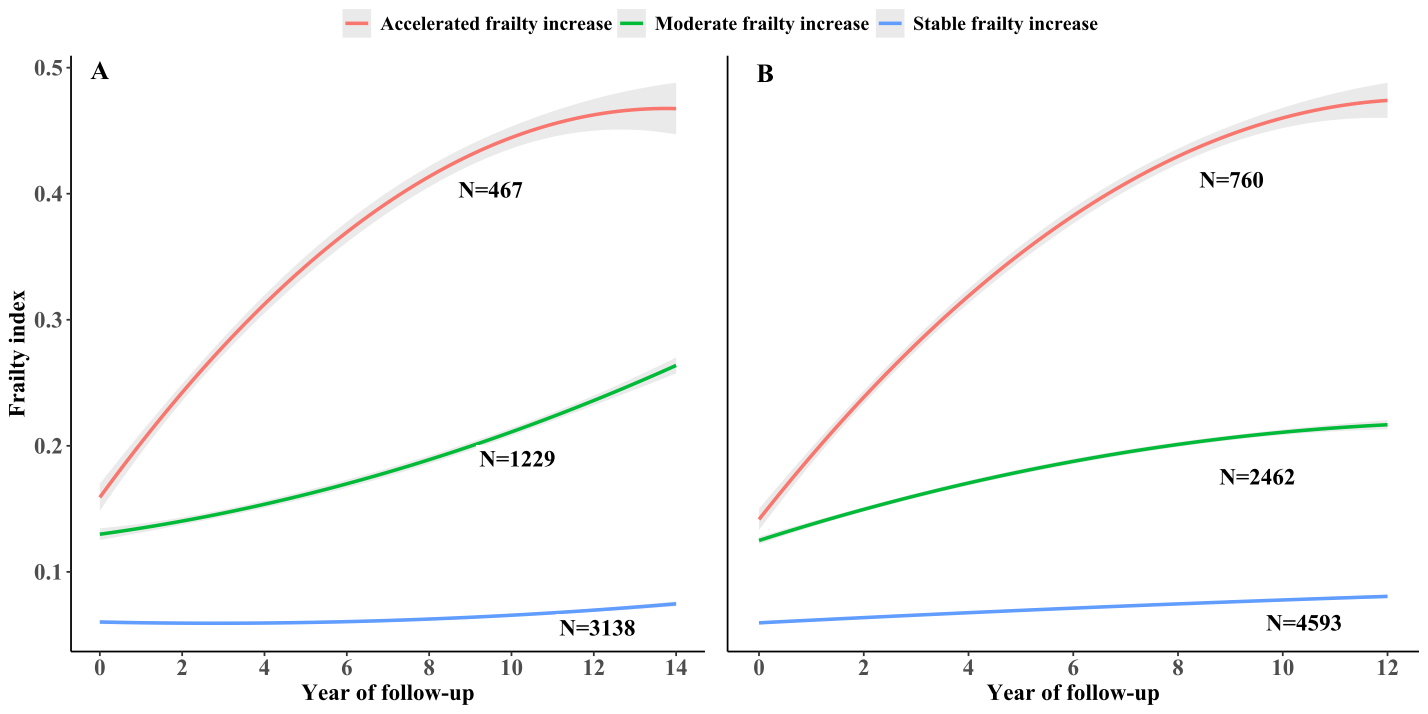


**Figure S3. Identified dynamic frailty trajectories using the group-based trajectory modeling approach in two independent cohorts, based on the modified 26-item (excluding hypertension, stroke, and diabetes) frailty index.**

Panel A: Frailty trajectories in the ELSA cohort; Panel B: Frailty trajectories in the HRS cohort.

**Figure S4. ROC analysis comparing the performance of predicting accelerated frailty increase by SCORE and SCORE2 in two independent cohorts, based on the modified 26-item (excluding hypertension, stroke, and diabetes) frailty index.**

Panel A: ROC analysis in the ELSA; Panel B: ROC analysis in the HRS.

ROC: receiver operating characteristic curve; AUC: area under the curve; the ELSA: The English Longitudinal Study of Aging; The HRS: The Health and Retirement Study; SCORE: Systemic Coronary Risk Estimation; SCORE2: Systemic Coronary Risk Estimation 2.


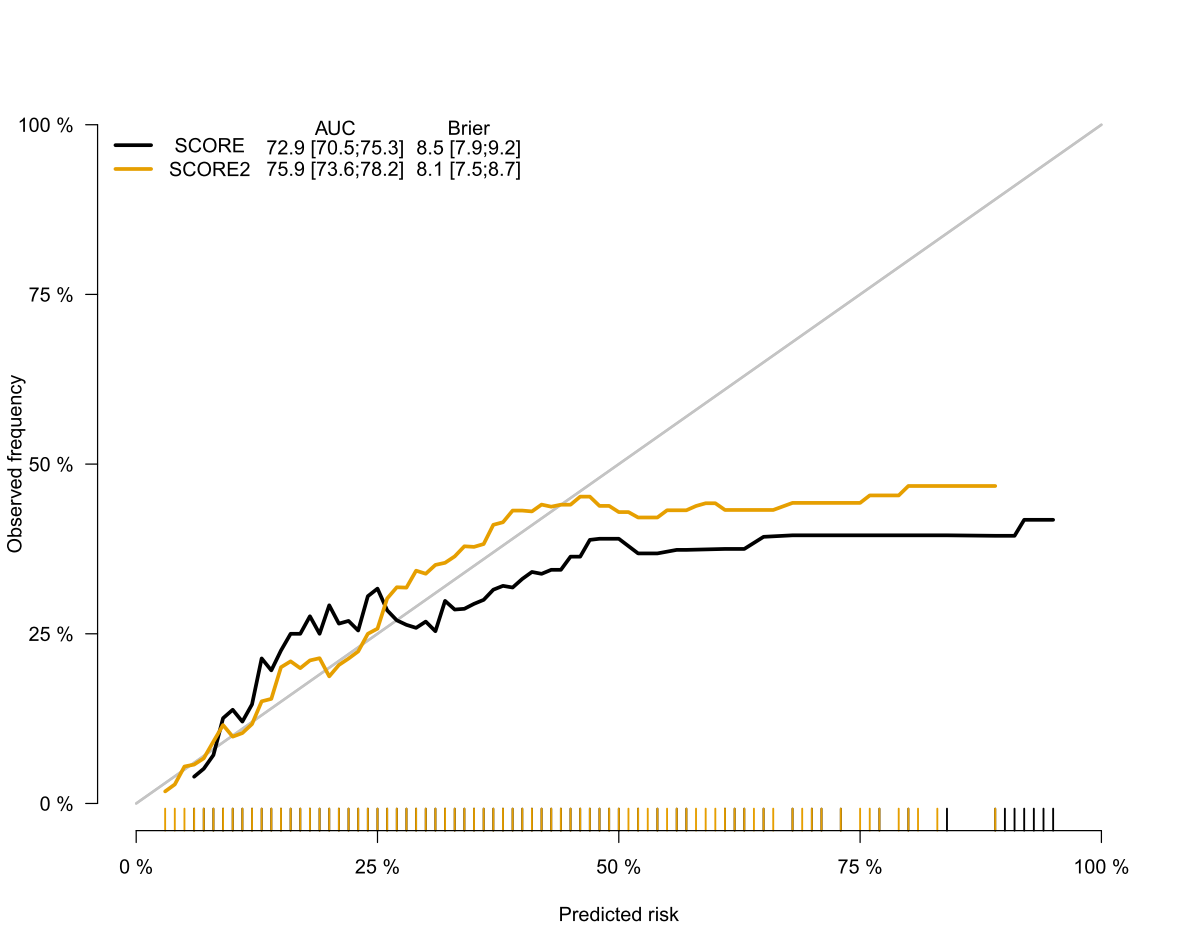


**Figure S5. Calibration plot comparing the performance of predicting accelerated frailty increase by SCORE and SCORE2 in the ELSA cohort.**


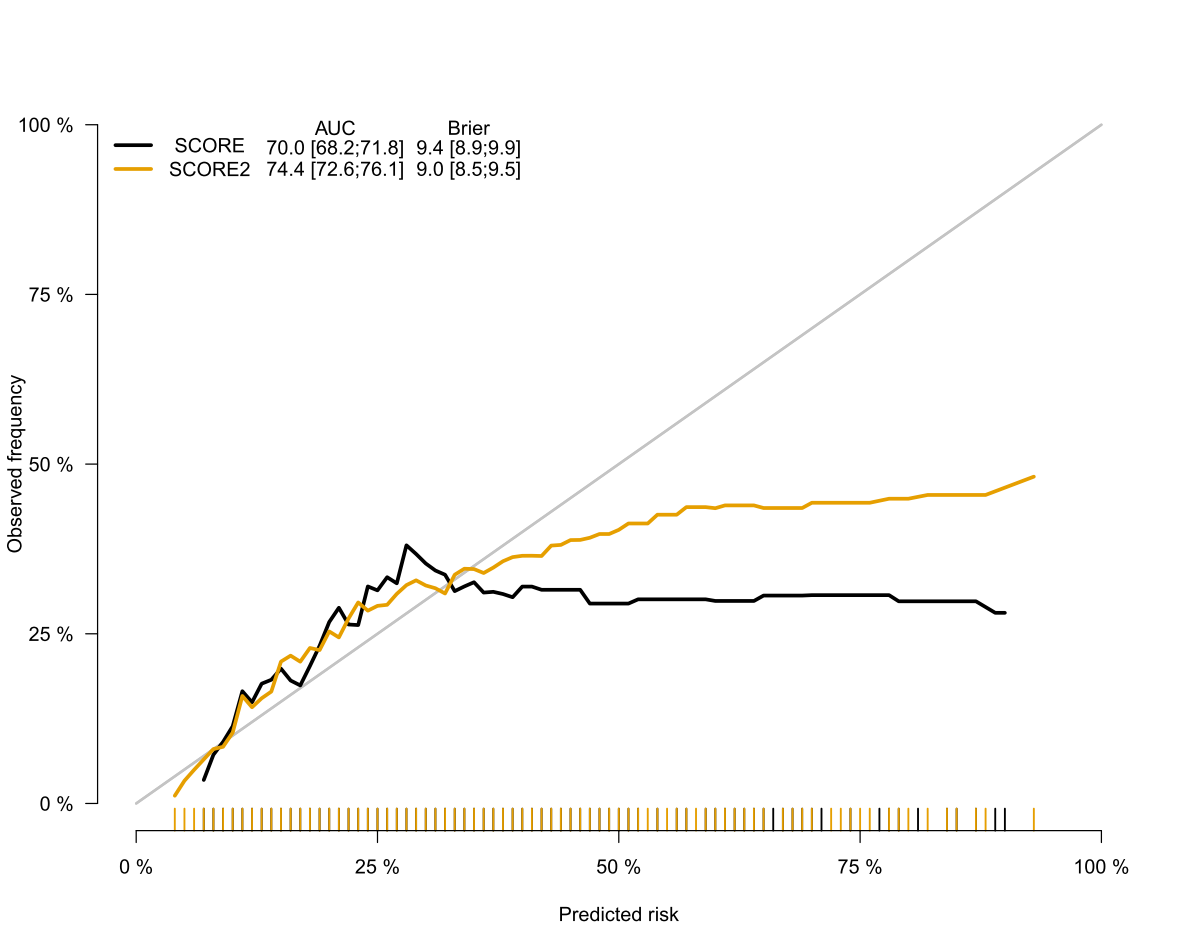


**Figure S6. Calibration plot comparing the performance of predicting accelerated frailty increase by SCORE and SCORE2 in the HRS cohort.**


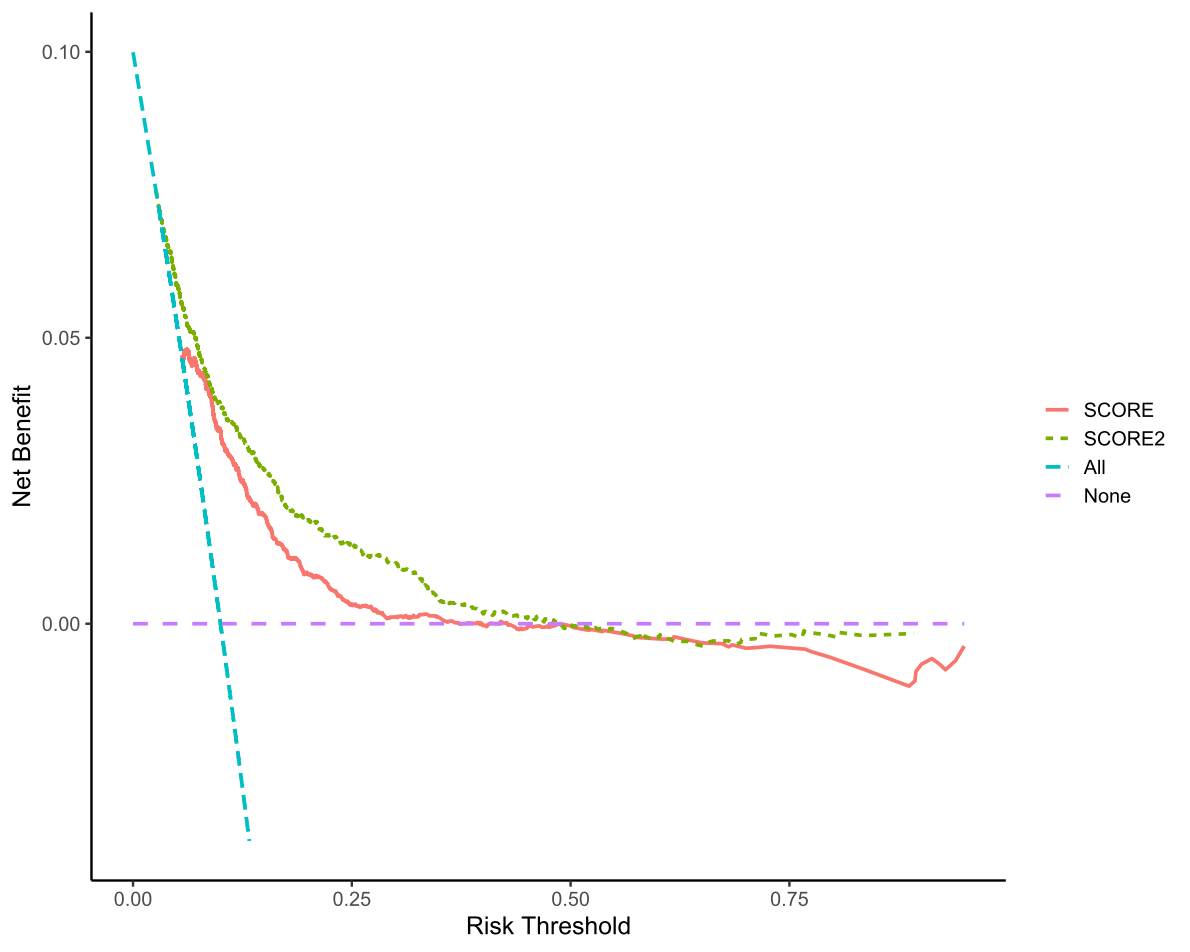


**Figure S7. Decision curve analysis for assessing the risk threshold of predicting accelerated frailty increase by SCORE and SCORE2 in the ELSA cohort.**


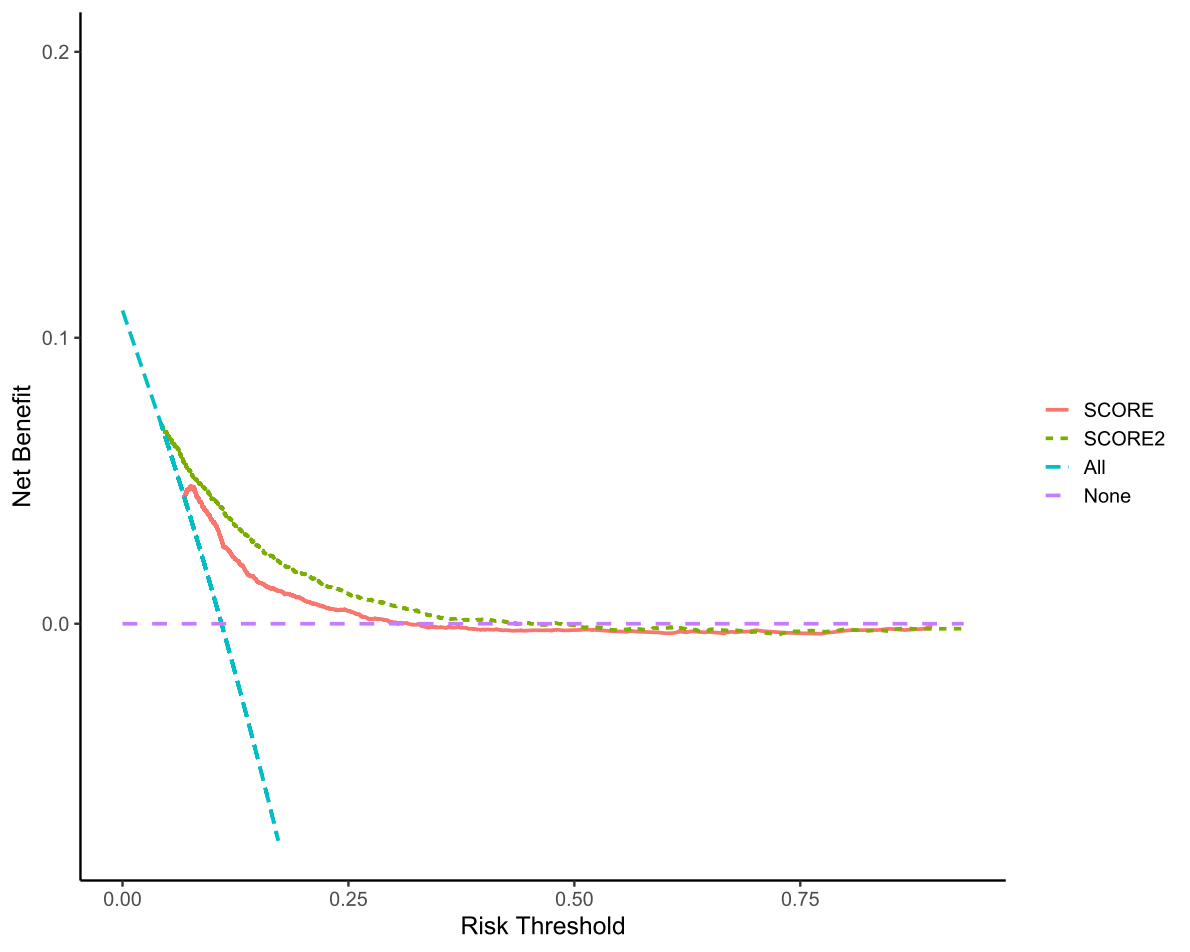


**Figure S8. Decision curve analysis for assessing the risk threshold of predicting accelerated frailty increase by SCORE and SCORE2 in the HRS cohort.**


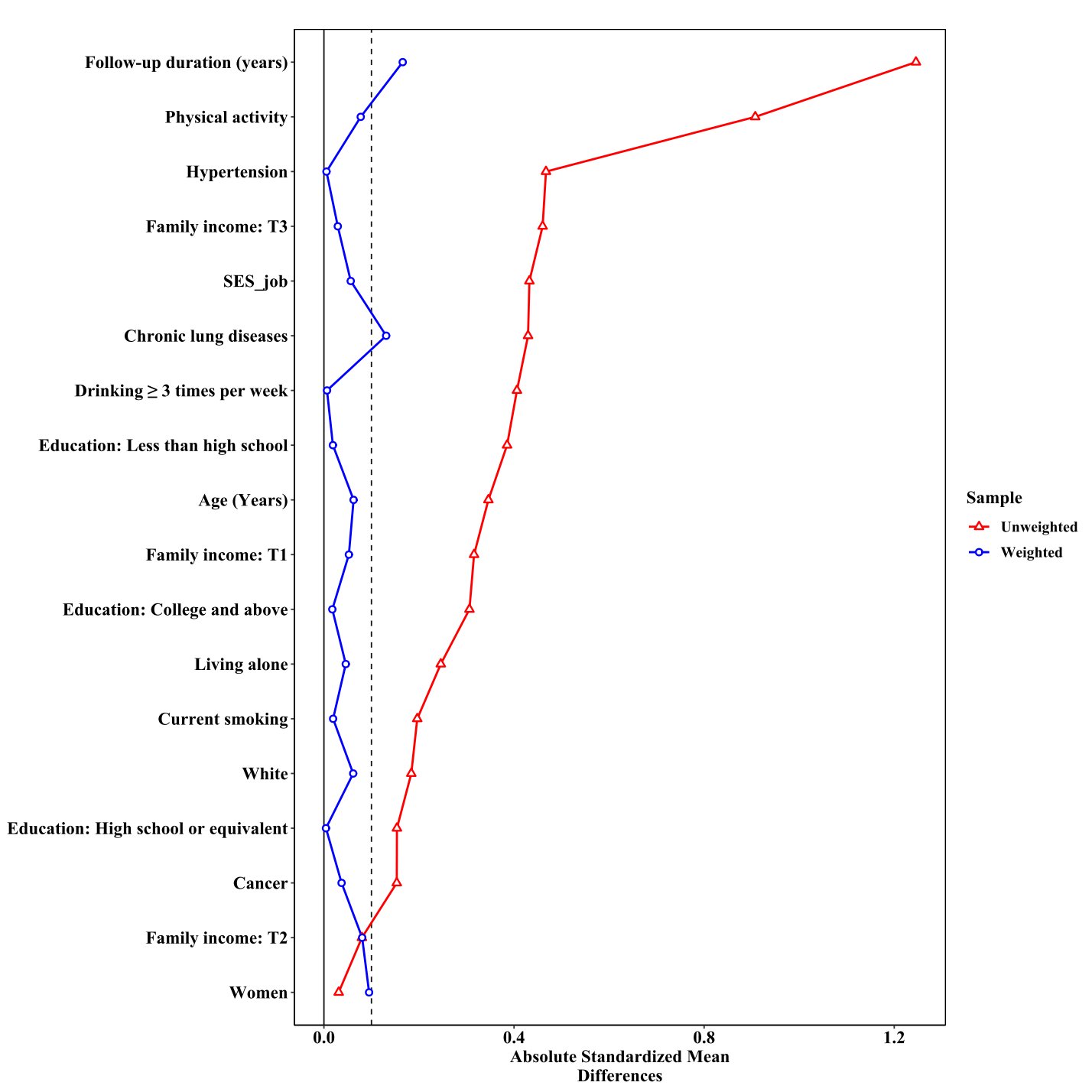


**Figure S9. Love plot assessing balance between included and excluded participants in the ELSA cohort, before and after the IPW weighting.**


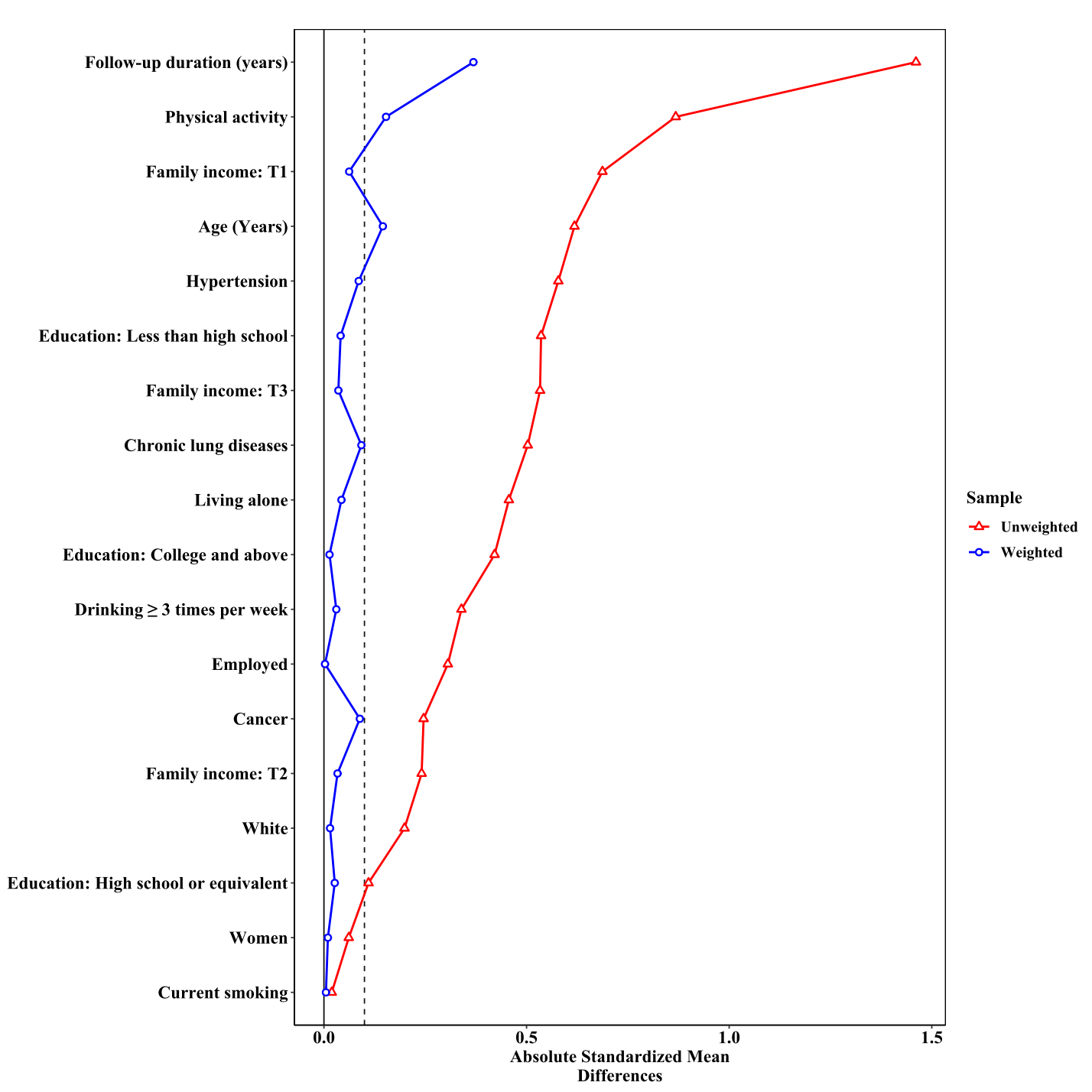


**Figure S10. Love plot assessing balance between included and excluded participants in the HRS cohort, before and after the IPW weighting.**


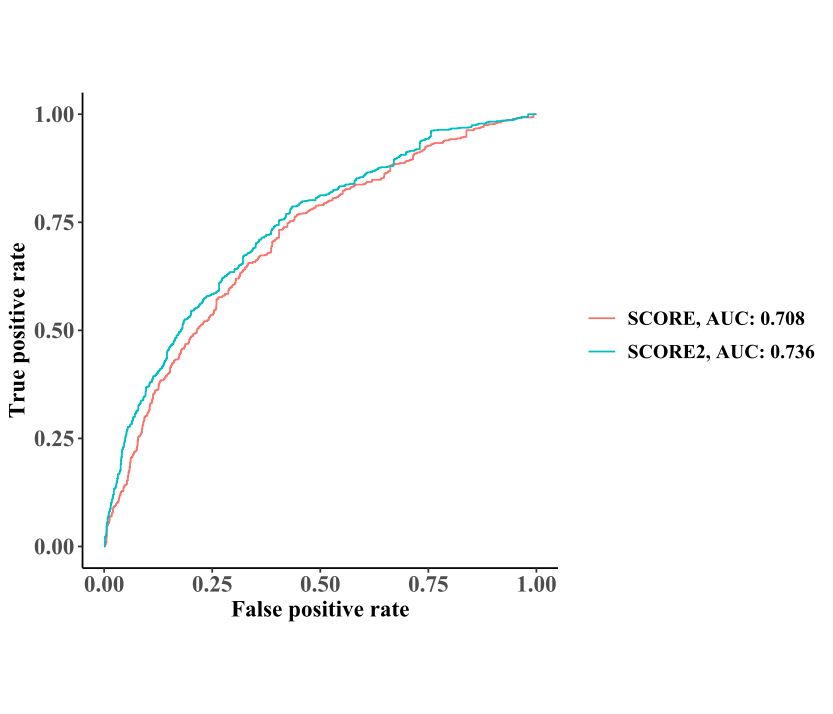


**Figure S11. Weighted ROC analysis comparing the performance of predicting accelerated frailty increase by SCORE and SCORE2 in the ELSA cohort, using the IPW as sample weights.**


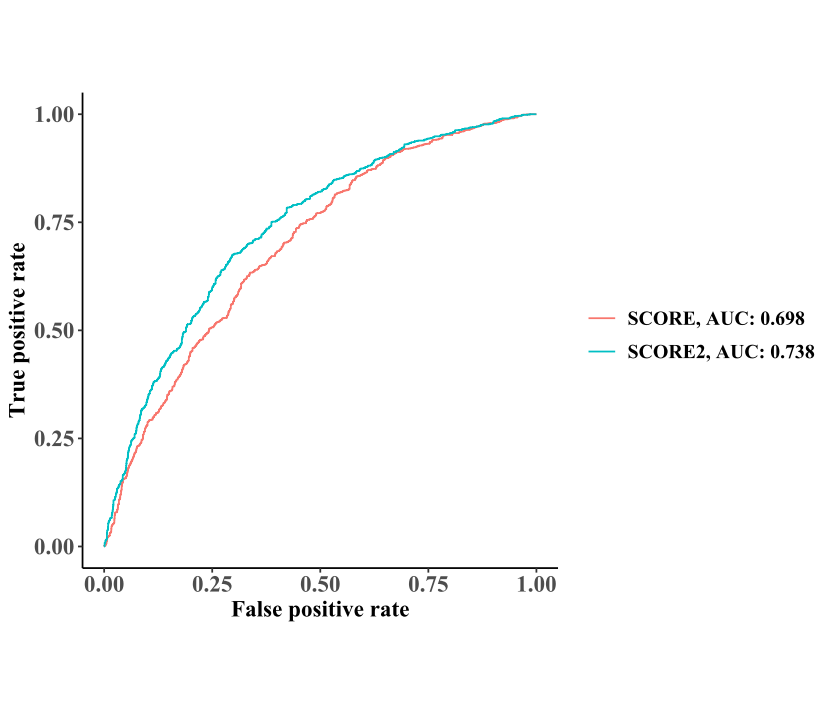


**Figure S12. Weighted ROC analysis comparing the performance of predicting accelerated frailty increase by SCORE and SCORE2 in the HRS cohort, using the IPW as sample weights.**

**Figure S13. ROC analysis comparing the performance of predicting moderate frailty increase by SCORE and SCORE2 in two independent cohorts.**

Panel A: ROC analysis in the ELSA; Panel B: ROC analysis in the HRS.

ROC: receiver operating characteristic curve; AUC: area under the curve; the ELSA: The English Longitudinal Study of Aging; The HRS: The Health and Retirement Study; SCORE: Systemic Coronary Risk Estimation; SCORE2: Systemic Coronary Risk Estimation 2.

**Figure S14. ROC analysis comparing the performance of predicting accelerated frailty increase by SCORE and SCORE2 in two independent cohorts, restricted to individuals aged < 65 years.**

Panel A: ROC analysis in the ELSA; Panel B: ROC analysis in the HRS.

ROC: receiver operating characteristic curve; AUC: area under the curve; the ELSA: The English Longitudinal Study of Aging; The HRS: The Health and Retirement Study; SCORE: Systemic Coronary Risk Estimation; SCORE2: Systemic Coronary Risk Estimation 2.

**Figure S15. ROC analysis comparing the performance of predicting accelerated frailty increase by SCORE and SCORE2 in two independent cohorts, restricted to individuals aged ≥ 65 years.**

Panel A: ROC analysis in the ELSA; Panel B: ROC analysis in the HRS.

ROC: receiver operating characteristic curve; AUC: area under the curve; the ELSA: The English Longitudinal Study of Aging; The HRS: The Health and Retirement Study; SCORE: Systemic Coronary Risk Estimation; SCORE2: Systemic Coronary Risk Estimation 2.
